# Can We Trust Measures of Political Trust? Assessing Measurement Equivalence in Diverse Regime Types

**DOI:** 10.1007/s11205-016-1400-8

**Published:** 2016-07-04

**Authors:** Irena Schneider

**Affiliations:** Department of Political Economy, Faculty of Social Science and Public Policy, King’s College London, Second Floor, Strand Building, Strand Campus, London, WC2R 2LS UK

**Keywords:** Political trust, Regimes, Measurement equivalence, Multiple group confirmatory factor analysis

## Abstract

Do standard “trust in government” survey questions deliver measures which are reliable and equivalent in meaning across diverse regime types? I test for the measurement equivalence of political trust in a sample of 35 former Soviet and European countries using the 2010 Life in Transition Survey II conducted by the World Bank and European Bank for Reconstruction and Development. Employing multiple group confirmatory factor analysis, I find that trust perceptions in central political institutions differ from (1) trust in regional and local political institutions, (2) trust in protective institutions like the armed forces and police and (3) trust in order institutions like the courts and police. Four measurement models achieve partial metric invariance and two reach partial scalar invariance in most countries, allowing for comparisons of correlates using latent factors from each model. I also found some clustering of measurement error and variation in the dimensionality of political trust between democratic and autocratic portions of the sample. On some measurement parameters, therefore, respondents in diverse cultures and regime types do not have equivalent understandings of political trust. The findings offer both optimism and a note of caution for researchers using political trust measures in cross-regime contexts.

**Electronic supplementary material:**

The online version of this article (doi:10.1007/s11205-016-1400-8) contains supplementary material, which is available to authorized users.

## Introduction

Since the 1960s and 70s, theorists have claimed that political trust is fundamentally important for democracy and political order. Rosanvallon (2008, 48–49) describes trust as an “invisible institution” or “assumed stock of information”, an essential “property of a relationship between…governors and governed” in which a “politician’s reputation becomes his certificate of warranty.” Political trust allows political authorities to provision public goods to the electorate without resorting to repression or coercion (Parsons 1961, 53; Luhmann 1979, 56). Declines in political trust across advanced democracies in the postwar era have been interpreted as a deterioration of state legitimacy (Easton 1975; Luhmann 1979) and even a “crisis of democracy” (Huntington et al. 1975). Recent research shows that low political trust levels are associated with tax fraud and low compliance with the law (Hooghe and Marien 2010) as well as low generalized trust and social capital (Rothstein 2003; Schyns and Koop 2010). The idea that political trust is important for democracy or good governance motivates a great deal of empirical research on developed democracies in Europe and the US (Hetherington 1998; Lipset and Schneider 1983; Marien 2011) and non-democracies such as China and Russia (Lovell 2001; Yang and Tang 2010). Examining former Soviet political transitions, for instance, Bowser (2001, 17) argues that “if the countries of the FSU [Former Soviet Union] are to proceed to genuinely representative and accountable democratic regimes they must seek to increase public support for the state.”

Although there is considerable consensus about the importance of political trust, there is little consensus about its definition or measurement. Hooghe (2011, 270) criticizes researchers’ dependence on standard “trust in government” survey questions “without questioning their validity or even wondering what political trust actually refers to, or what place the concept could have in democratic society.” Researchers do not usually propose clear definitions of political trust but often take it to be a proxy for political legitimacy (Almond and Verba 1963; Anderson and Tverdova 2003; Chang and Chu 2006; Christensen and Laegreid 2005; Coromina and Davidov 2013; Hooghe 2011; Hutchison and Johnson 2011; Kim and Voorhees 2011; Mishler and Rose 2001; Newton 2007; Suh et al. 2012). A measure of political trust carries implicit information about what constitutes a trustworthy institution for citizens. Beetham (2013, 11) defines political legitimacy as an “assessment of the degree of congruence, or lack of it, between a given system of power and the beliefs, values and expectations that provide its justification.” Hetherington (1998, 791) conceives of political trust in a similar way, describing it as “a basic evaluative orientation toward the government founded on how well the government is operating according to people’s normative expectations.”

We are, in principle, dealing with a very important and normatively charged concept which is increasingly being studied in all parts of the world. Yet researchers continue to rely on sum scores or averages of standard “trust in government” survey questions without fully understanding what the concept means, or whether these measures tap into comparable ideas across the countries in their sample. Precisely because of the normative and subjective content of political trust, what constitutes a trustworthy institution is unlikely to be the same for citizens in different cultural and regime contexts. Moreover, cross-national research in more diverse environments enhances potential for measurement error resulting from the data collection process with potentially detrimental consequences for regression analysis. Prior to comparing the means or correlates of political trust survey indicators, it is important to check that the indicators deliver similar and comparable understandings of political trust across populations. Little progress has been made in this direction even though our ability to accurately theorize about the causes and consequences of political trust depends on these empirical considerations.

To account for this weakness in the comparative literature, I aim to answer two questions about the measurement of political trust in 35 European and former Soviet countries using the 2010 Life in Transition II Survey (LITS II). First, do standard “trust in government” survey indicators represent a single, comprehensive attitude of political trust? Second, are different measurement models of political trust equivalent in all countries? In other words, do measures of political trust travel successfully on the survey instrument across borders? I will investigate these questions using multiple group confirmatory factor analysis (Jöreskog 1971). Unlike most studies of the measurement equivalence of political trust, the results of this study will help us determine our ability to compare the means and correlates of the construct across both democracies and non-democracies.

## Empirical Considerations

Data limitations are inevitable in the measurement of political trust. Most surveys do not contain indicators which capture elements of institutional trustworthiness for a given population. As a result, scholars typically produce sum scores or averages of survey indicators measuring trust in a variety of political institutions on a likert scale (from strong distrust to strong trust). Usually, little to no rationale accompanies these item choices. To consider just a few examples from highly cited studies, Mishler and Rose (2001) examine the sources of political trust in ten post-communist societies surveyed in the New Democracies Barometer by averaging trust in the parliament, prime minister or president, courts, police, parties and military. Chang and Chu (2006) and Chang (2013) use the East Asian Barometer to estimate the effect of corruption on political trust in six Asian countries by averaging trust in the president/prime minister, courts, national government, political parties, parliament, civil service, military, police and local government. To assess the importance of political capacity for political trust in environments with different levels of violence, Hutchison and Johnson (2011) construct an additive index of trust in the executive, courts, police, armed forces, electoral commissions and government-run media for 16 countries surveyed in the Afrobarometer. Clausen et al. (2011) use the Gallup World Poll to study political trust and corruption in 103 countries, obtaining an index of confidence in public institutions by summing responses to a question on confidence in the military, judicial system and courts, national government and honesty of elections. Similar measurement approaches are taken in cross-national research projects on political trust in Latin America (Seligson 2002; Stoyan et al. 2014), Asia (Wong et al. 2011), Sub-Saharan Africa (Cho et al. 2007; Lavallée et al. 2008) and other global samples (Catterberg and Moreno 2005; Hakhverdian and Mayne 2012).

This prolific “kitchen sink” measurement approach is a problem if our goal is to draw meaningful inferences about political trust across diverse societies. Comparing the correlates of averaged or summed indicators across countries assumes that these indicators are (1) reliable, unidimensional measures of political trust in each country and (2) mean the same thing to respondents in each country. Could we assume, for instance, that citizens on the democratic and autocratic parts of the regime spectrum have similar understandings of what it means for an election to be honest? Can we be sure that fear to report true beliefs about the executive is not biasing the responses of a person affected by a violent civil war? Do citizens of East Asian countries really evaluate the president and police in similar ways? Perhaps petty corruption might reduce trust in the police but not in a charismatic president.

While testing for the validity and equivalence of empirical measures is common practice in psychology and management studies, it is less recognized in political science (Adcock and Collier 2001, 536; Ansolabehere et al. 2008, 228). Measurement error in survey research can result from inherently different understandings of survey questions across different populations, as well as from method effects specific to the survey instrument. Survey implementation, translation, and question order can influence nonresponse patterns, uses of extreme response categories and socially desirable responses by population (Davidov et al. 2014, 59–62; Podsakoff et al. 2012, 544). In applied research, cross-cultural comparisons of attitudes toward democracy, levels of postmaterialism and left–right political ideology, for instance, do not pass the test of measurement equivalence (Alemán and Woods 2015; Davidov et al. 2014). Delhey et al. (2011) find significant cross-national variation in the way respondents interpret “generalized trust” in the World Values Survey by estimating their “trust radius”, or the width of one’s notion of trust in “most” people. They find that the trust radius is much smaller for people in countries with Confucian influence than for those in countries with a Protestant heritage and modern economy, noting that such findings “throw sufficient doubt on the cross-national validity of the standard trust question” (ibid, 793).

Measurement testing procedures are only recently appearing in political trust research. Hooghe (2011) uses factor analysis to show that British citizens do not distinguish between MPs, governing parties, opposition parties or the head of state regardless of political sophistication or education. Suh et al. (2012, 516) demonstrate in a latent class analysis that trust in government is part of a broader set of attitudes towards public and private institutions like companies and civil associations in South Korea. A number of studies implement a multiple group confirmatory factor analysis (MGCFA) of political trust models in the European Social Survey (ESS), all finding relatively strong evidence for the equivalence of political trust across subsets of countries and time points. The authors’ choices of indicators, however, are not theoretically consistent.

Allum et al. (2011, 42), for instance, use only trust in the parliament and politicians, arguing that a prior confirmatory factor analysis (CFA) showed that trust in the legal system, police, European parliament and UN constituted a separate dimension of trust. Coromina and Davidov (2013, 41), however, choose trust in the parliament, legal system and politicians using the same survey. They do not justify this precise combination of indicators even though they acknowledge that legal institutions like courts are often conceptualized separately from strictly political institutions because they are meant to be impartial and focused on enforcing the rule of law (Jackson et al. 2011; Linde 2012; Rothstein and Stolle 2008). Marien (2011) chooses the broadest set of indicators from the ESS, including trust in the parliament, politicians, political parties, legal system and police, detecting an error correlation between trust in the police and legal system. This finds some confirmation in Schaap and Scheepers’ (2014) assessment of the measurement equivalence of trust in police in 27 European countries. André (2014) also uses a broad range of indicators in the ESS to test for the equivalence of political trust between EU natives and migrants, but introduces three correlated errors to illustrate the multidimensionality of the construct as distinctively political (measured by trust in politicians, parliament and political parties), order/neutral (trust in the legal system and police) and international (trust in the EU parliament and United Nations).

Although these papers have different theoretical purposes, it is striking how many different models of political trust can fit mostly the same data in a single region of the world. A cross-regime survey will likely invite even more variation in indicator selection and increase the potential for measurement error across countries. There is, however, no substitute for conscientious measurement modeling in regression analysis. Feeding unreliable and non-equivalent measures into a pooled regression can undermine the validity of substantive results. Reeskens and Hooghe (2008, 527) and Coromina and Davidov (2013, 48) show that using country means without accounting for measurement error and equivalence results in incorrect country rankings on social and political trust. Bertrand and Mullainathan (2001, 70) go as far as to argue that “subjective variables cannot reasonably be used as dependent variables, given that the measurement error likely correlates in a very causal way with the explanatory variables.” If a sum score of political trust is used as a dependent variable, for example, predictors might not ultimately be explaining variation in political trust scores, but rather the way those scores are over or under-reported. Westfall and Yarkoni (2016, 12) and van der Veld and Saris (2011, 241) demonstrate that multiple regression capitalizes on measurement error by incorrectly apportioning the amount of explained variance in the dependent variable between different error-laden predictors. Both authors recommend structural equation modeling (SEM) to control for measurement error and thus more accurately determine each predictor’s effect on the outcome.

Ultimately, though political trust is considered an important object of study, it currently rests on a weak theoretical and empirical foundation. Neglecting the criteria for empirical measurement can undermine our ability to draw meaningful and accurate inferences about substantive theories using regression analysis. While this issue is gaining traction in political trust research, most tests of measurement equivalence remain limited to Europe and specifically to the European Social Survey. Techniques like MGCFA have not yet enriched measurement modeling in developing and authoritarian countries where survey research has proliferated in the last decade. To help overcome this weakness, I will put to test the ability of different measurement models of political trust to meet the requirements of validity and equivalence across different cultures and regime types.

## Case Selection and Data

Considerable region-specific research on political trust outside of Europe and the United States has covered parts of Asia, Africa, Latin America, and selections of contemporary and developing democracies. Political trust in parts of the former Soviet space has received some empirical attention (Luhiste 2006; Mishler and Rose 1997, 2001; Wallace and Latcheva 2006), although these regional samples have neglected countries in the Southern Caucasus and Central Asia mainly due to a lack of data.

In this study I use the Life in Transition Survey II (LITS II) produced by the European Bank for Reconstruction and Development (EBRD) and the World Bank. The cross-sectional sample from late 2010 surveys almost 39,000 households in thirty-five countries to assess public attitudes on a range of social, political and economic variables. This sample contains all former Soviet countries, the Balkans, Eastern and Central Europe, and some of Western Europe.^1^ Two –stage clustered, stratified sampling was employed across regions in each country.^2^ 38,379 response observations (with at least 895 per country) are used in this analysis due to missing values on all variables in 485 observations. Respondents were asked “To what extent do you trust the following institutions?” (including the presidency/monarchy, government/cabinet of ministers, regional government, local government, parliament, courts, political parties, armed forces, police) using a 1–5 likert scale (from complete distrust to complete trust).

Helpfully, this survey includes Central Asia and the Southern Caucasus, allowing for systematic comparison between significantly different cultures and regime types. The inclusion of these rarely-explored regions introduces striking variation on trust perceptions into the sample. Looking only at the single ‘trust in government’ indicator without controlling for measurement error, the most authoritarian countries appear to be the most trusting of government with the exception of Sweden (Uzbekistan exhibiting the most trust, followed by Tajikistan, Sweden, Azerbaijan, Kazakhstan, Turkey, Belarus, Georgia, Montenegro and Russia). On this basis it could be argued that subjecting these indicators to cross-national comparison inappropriately assumes that the institutions in question have sufficiently similar roles and functions. After all, parliaments and political parties can be considered fundamentally dysfunctional in autocracies like Uzbekistan, and thus incomparable to the same institutions in Sweden.

While it is true that a host of political institutions are functionally dissimilar in democracies and autocracies, the parameter on which comparisons are drawn in the present case (as in much of the political trust research) is based on perceptions, rather than observable attributes of institutions. In testing the validity of a perception-based measure, we must ensure that we are working with approximately similar associations of political trust among respondents in multiple locations. If, say, the parliament proves to be different enough in two countries so as to inspire inherently different understandings of its purposes and functions, this deviation can be detected by measurement equivalence testing. Likewise, biases in response arising from fear to reveal genuine opinions or misinterpretations based on faulty question translations or interview techniques may also lead to statistical nonequivalence. A comparison between countries on the indicator would be deemed un-interpretable in these cases. If, however, despite significant differences in institutional functionality attitudes across countries refer to the same approximate idea, we can proceed with comparisons on the perception-based measures even in diverse regime contexts.

## Analytical Strategy

I use Multiple Group Confirmatory Factor Analysis (MGCFA), a powerful statistical tool in the family of structural equation modeling commonly used to assess the measurement equivalence of a latent construct across populations. MGCFA is typically employed after valid measurement models have been specified in all groups (in this case, countries) either via exploratory factor analysis (EFA) or a strong theoretical foundation, and tested for appropriate “goodness of fit” to survey response data via confirmatory factor analysis (CFA). More specifically, the purpose of EFA is to explore inter-correlations among a set of indicators to generate the smallest number of unique factors that can best explain these correlations (Brown 2006, 20). In CFA, rather than simply exploring data for sets of patterns, the researcher uses theoretical reasoning to make a priori specifications of the measurement model, constraining specific indicators to load on specific factors. This procedure tests the validity of the researcher’s measurement model by showing how much the model specification adheres to covariance patterns in the survey data.

Measurement equivalence is used interchangeably with the statistical term “invariance,” which refers to “whether or not, under different conditions of observing and studying phenomena, measurement operations yield measures of the same attribute” (Horn and McArdle 1992, 117; Steenkamp and Baumgartner 1998, 78). Conventionally, three stages of invariance must be achieved before the comparison of means can occur. When testing for *configural invariance*, we want to understand whether the same survey indictors measure the same latent construct in all groups. Reaching this level means that the same basic meaning and structure of political trust exists in all countries. *Metric invariance* refers to the equality of factor loadings on the construct across groups. A unit change in the latent factor of political trust will affect scores on political trust survey questions by the same magnitude across countries. Reaching this level of invariance is sufficient for meaningful cross-cultural comparisons of covariances and unstandardized regression coefficients. That is, we can be confident that changes in political trust scores arise from real differences in the underlying construct rather than nuisance variables or method effects (Byrne et al. 1989; Millsap 2011). Finally, *scalar invariance* refers to the equality of intercepts across groups. In this case, differences in indicator means result from differences in latent factor means (Steenkamp and Baumgartner 1998, 80). Although this level of invariance is typically required to meaningfully compare factor means across groups, *partial scalar invariance* is generally considered sufficient if at least two indicators per factor have invariant loadings and intercepts in each group (Byrne et al. 1989; Byrne 2012, 198; Brown 2006, 81–82). While respondents might understand survey questions similarly (given metric invariance), it may still be problematic to compare means if the model fails to achieve partial scalar invariance.

I follow the literature in assuming that political trust indicators *reflect* a broader attitude toward political institutions; they do not *generate* it like education, income and occupation generate the concept of socioeconomic status. Indicators like trust in the parliament, political parties and prime minister have been shown to be highly correlated and interchangeable in the CFA and MGCFA literature, which is the opposite of what one would expect of a formative or ‘generating’ approach to index construction. In the latter approach, items should have a distinct influence on the measured construct in a way that it would lose substantive meaning without each item. High inter-correlations among items would signal redundancy and multicollinearity rather than reliable internal consistency (Diamantopoulos and Siguaw 2006, 267). It would be hard to argue that any single political trust indicator adds a distinct or essential contribution to a person’s broader political orientation. More realistically, political culture inside a country affects how one relates to a number of political institutions (Marien 2011, 17). The arrow of influence thus moves from the latent, unobserved dimension of political trust to one’s position on the observed indicators.

## Constructing Four Measurement Models

Due to the sheer number of survey items used in the literature without a theoretical rationale, I consider different plausible measurement models of political trust. I begin with a simple exploratory factor analysis (EFA) in each country using a broad range of commonly used indicators. From these solutions and some theoretical consideration, I construct four measurement models of political trust and subject them to tests of measurement validity and equivalence. While the EFA solutions are no substitute for theory, they strongly suggest that indicators as diverse as trust in the government, parliament, parties, police, armed forces and courts do *not* form a unidimensional model of political trust in most countries.^3^

Firstly, EFA output for approximately half of the countries suggests that a separate factor accounts for trust in regional and local political institutions, indicating that many citizens differentiate between local and federal levels of government. To test for this possibility, I construct Model 1 by specifying trust in the government, parliament, political parties, regional and local government to load on a “political trust” latent variable, adding an error correlation between trust in the regional and local government. While the survey is unclear about what “regional” and “local” politics entail, it is likely that many respondents associate “regional” politics with the *rayon*, a Soviet-era administrative division of government slightly below the federal level which many (though not all) states retained after the collapse of the Soviet Union. The local level will likely correspond to city and town districts closer to the individual. Some variation might be expected between countries which inherited this structure from the Soviet Union and those which did not in Eastern or Central Europe. In Bulgaria, for instance, the *rayon* refers to a city-level rather than national government subdivision. If this model specification produces good fit to the data in most countries, we might conclude that local and regional political trust responses co-vary together in ways that cannot be accounted for by the political trust factor. At the same time, regional political trust might be more associated with federal-level political trust in former Soviet countries.

Secondly, EFA solutions show that citizens trust the police and armed forces in a different way than they trust political institutions like the government and parliament. Despite the lack of theory to support this dimension of political trust, the police and armed forces indicators form salient loadings on a separate factor in most countries in the sample. One possibility is that these institutions are the only ones which can legally exercise force to protect citizens. At least in principle, they may represent deeper notions of order that go beyond the tides of party politics, eliciting notions of patriotism or legitimacy which the parliament or government do not. To test for this “protective” dimensionality, I specify trust in the government, parliament, political parties, armed forces and police to load on a single factor, adding an error correlation between the latter two indicators. If this specification produces a good fit to the data in most countries, we can conclude that respondents think about the police and armed forces differently than they think about political institutions.

Thirdly, as mentioned earlier, many authors argue that people evaluate the courts and police differently from the government or parliament because they are meant to be impartial and devoted to the maintenance of the rule of law and criminal justice. My EFA solutions do not consistently support this argument partly because trust in the courts loads on factors accounting for trust in political institutions like the government and parliament in authoritarian former Soviet states. It is likely that people living under politically repressive regimes might not believe that judicial and political institutions are independent of each other. I test whether this is the case by specifying trust in the government, parliament, political parties, courts and police to load on one ‘political trust’ factor with an error correlation between the courts and police. This specification may produce poorer fit in more politically repressive contexts.

Because indicators involving courts, armed forces, police and regional government are subject to error correlations and separate dimensions of political trust, they are likely to cause problems for cross-national equivalence. In the interest of capturing the largest amount of countries for an appropriate cross-national regression or mean comparison, in Model 4 I specify a simple political trust factor measured by trust in the government, parliament, political parties and local government. This model is closest to other CFA models of political trust using the European Social Survey and should produce the best fit to the data in most countries.

**Fig. 1:**
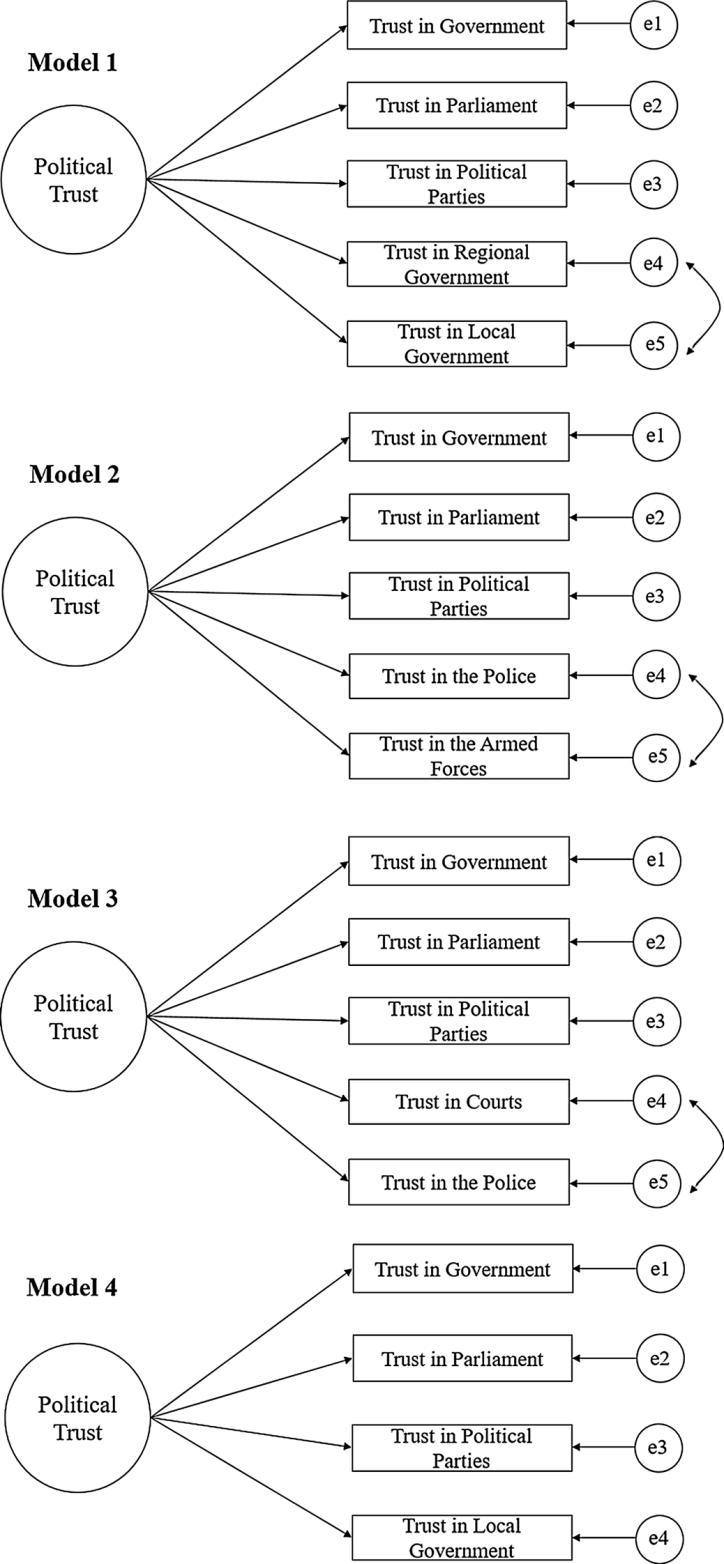
These diagrams represent linear factor models in which the latent (unobserved) political trust factor explains variation in observed indicators. Errors (*in small circles*) represent variation in observed indicators left unexplained by the latent factor. Correlated errors are represented by *curved arrows*, which indicate special covariances between indicators for theoretically specified reasons

For ease of interpretation, I have included path diagrams of each measurement model in Fig. 1. To check for the robustness of these models to alternative specifications, I compare each model to a bi-dimensional model in which the two items originally specified to have correlated errors are reflected by a separate factor. I also compare Model 4 to one excluding trust in the local government, keeping the factor strictly limited to federal-level political institutions. Across all models, I exclude the ‘trust in the presidency/monarchy’ indicator. British respondents would have been evaluating trust in the Queen, French respondents the French President and Belarusians an autocratic leader in power since 1994. A cross country comparison on such an indicator would be de-facto uninterpretable based on its heterogeneous content. This point finds confirmation in the EFA output, which does not show consistent factor loading patterns of the indicator across countries.

I begin with separate country CFAs and follow up with invariance testing on each model using Mplus 7. I run each model using Maximum Likelihood estimation, which takes into account all available data. When evaluating factor loadings, I consider a salient, standardized loading to be higher than 0.30 (Brown 2006). To assess each model’s goodness of fit, I use several global fit statistics, including the Chi square statistic, the RMSEA (root mean square error of approximation), the CFI (comparative fit index) and the SRMR (standardized root mean square residual). To reach acceptable fit, CFI should be greater than 0.95, SRMR below 0.10 and RMSEA below 0.08 (Schermelleh-Engel and Moosbrugger 2003). I evaluate local model fit by considering expected parameter changes (EPC), modification indices (MIs) and the power of the MI test using the JRule for Mplus package (Saris, Satorra and van der Veld 2011; Oberski 2009).^4^ Since I use ML estimation on large sample sizes in each country, I do not use the Chi square difference test to assess the extent of the degradation of model fit between different levels of invariance. The full output, including standardized factor loadings and fit statistics for each country in each model, is available in the Online Appendix.

## Results: Model 1

**Fig. 2:**
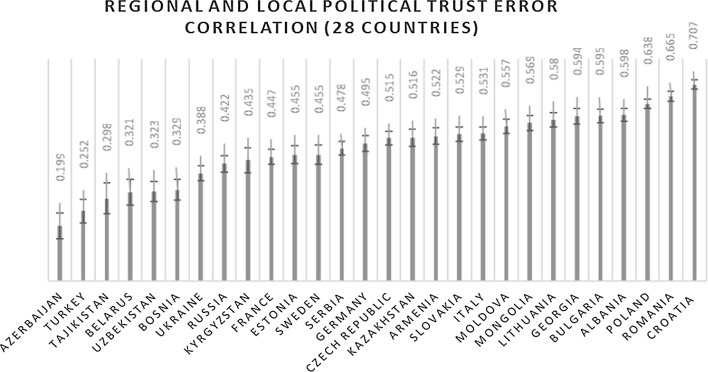
Countries are ranked by the ascending error correlation between regional and local political trust with 95 percent confidence intervals. This error correlation represents the proportion of shared variation between these indicators which cannot be explained by a political trust latent factor

In Model 1, trust in the government, parliament, political parties, local and regional government load on one factor with an error correlation between the latter two indicators. Due to missing information on the regional trust indicator, Great Britain, Hungary, Kosovo, Latvia, Macedonia, Montenegro and Slovenia were not included in the analysis. Off the bat, there appears to be interesting variation in the way the model behaves across the surveyed territory. I ordered the countries by the ascending error correlation between regional and local political trust (Fig. 2). In the bulk of the former Soviet countries alongside Bosnia and Turkey, the extent to which regional and local political trust have a special relationship that cannot be accounted for by the political trust factor is relatively small, but gets progressively bigger in Western and Eastern Europe.

Azerbaijan has the smallest error correlation (0.199), while Croatia has the largest (0.707). This means that local and regional political trust is decisively more related to trust in central political institutions in the former Soviet states than it is in Eastern and Western Europe. It is likely that regional and local political trust can load directly on a political trust factor without an error correlation in Azerbaijan, while in Croatia and select Eastern European countries, regional and local political trust might be modeled by a separate factor entirely. This could be due to different understandings of what constitutes “regional” politics or it could signify that citizens in former Soviet countries perceive that local and regional politics are subject to control by the federal center.

As expected, the same structure of the model is not present in several countries, preventing the model from reaching configural invariance across 28 countries. I use JRule to assist in detecting countries with the highest misspecifications. Several countries demonstrated more than one misspecification (for instance significant MIs recommend double error correlations between trust in the government and regional government, or political parties and parliament) which I chose not to correct for lack of a theoretical rationale. After removing Albania, Turkey, Kyrgyzstan, Russia, Tajikistan, Bulgaria and Estonia on these grounds, the model was able to achieve borderline configural invariance in 21 countries (chi sq = 756.971, DF = 84, RMSEA = 0.086, CFI = 0.990, SRMR = 0.014). These 21 countries are Armenia, Azerbaijan, Belarus, Bosnia, Croatia, Czech Republic, France, Georgia, Germany, Italy, Kazakhstan, Lithuania, Moldova, Mongolia, Poland, Romania, Serbia, Slovakia, Sweden, Ukraine and Uzbekistan. Though RMSEA is a bit higher than desired, JRule shows no misspecifications.

The model reached metric invariance with mostly acceptable fit statistics. After I freely estimated loadings for trust in political parties in Belarus, Uzbekistan and Georgia, trust in government in Azerbaijan and trust in local government and parliament in France, the model achieved partial metric invariance (chi sq = 1244.946, DF = 158, RMSEA = 0.079, CFI = 0.985, SRMR = 0.059). As expected, model fit deteriorated in the scalar invariance test. After I relaxed equality constraints on intercepts for trust in the government, local government and political parties in all countries and released one more factor loading constraint in four countries, the model achieved borderline acceptable partial scalar invariance (chi sq = 1910.107, DF = 174, RMSEA = 0.096, CFI = 0.975, SRMR = 0.074). Standardized factor loadings are substantial and significant in all countries. I have reported unstandardized factor loadings on the partial scalar model in Table 5. It is possible to compare correlates of political trust across countries using this latent factor. Even mean comparisons are possible with some caution (Table 1).

**Table 1:** Model 1: error correlation between regional and local government (21 countries) Data source EBRD and World Bank. All available data are used in ML estimation

	Chi square	DF	RMSEA	CFI	SRMR
Configural	756.971	84	0.086	0.990	0.014
Metric	1456.574	164	0.085	0.982	0.073
Partial metric	1244.946	158	0.079	0.985	0.059
Scalar	3709.260	238	0.116	0.951	0.107
Partial scalar	1910.107	174	0.096	0.975	0.074
*Compare: bi*-*dimensional model (regional and local trust reflect separate factor, 21 countries)*					
Configural	756.968	84	0.086	0.990	0.014
Metric	1123.686	144	0.079	0.986	0.048
Scalar	2394.798	204	0.099	0.969	0.059
Partial Scalar	1878.912	184	0.092	0.976	0.057

*DF* degrees of freedom, *RMSEA* root mean square error of approximation, *CFI* comparative fit index, *SRMR* standardized root mean square residual

I compared this model to a bi-dimensional model in which trust in the government, parliament and political parties is reflected by one factor and trust in the regional and local government is reflected by a second factor. It is reasonable to test the validity of this model particularly because several countries in the sample have a very high error correlation between local and regional trust. This model also reaches borderline partial scalar invariance with slightly better fit statistics across the same 21 countries (chi sq = 1878.912, DF = 184, RMSEA = 0.092, CFI = 0.976, SRMR = 0.057). Comparisons of correlates and cautious comparisons of means are also possible using this bi-dimensional measurement model of political trust.

## Results: Model 2

In Model 2, trust in the government, parliament, political parties, armed forces and police load on one factor with an error correlation between the latter two indicators. In this “protective” trust model, I test whether respondents distinguish between strictly political institutions and institutions which can use force to offer protection. Using JRule, I identified countries with multiple misspecifications (Belarus, Uzbekistan, Kyrgyzstan, Bulgaria, Kazakhstan and Estonia) and removed them before the model could reach configural invariance with 29 countries (chi sq = 692.945, DF = 116; RMSEA = 0.067, CFI = 0.991, SRMR = 0.020). These 29 countries are Albania, Armenia, Azerbaijan, Bosnia, Croatia, Czech Republic, Macedonia, France, Georgia, Germany, Hungary, Italy, Kazakhstan, Kosovo, Kyrgyzstan, Latvia, Lithuania, Moldova, Mongolia, Montenegro, Poland, Romania, Russia, Serbia, Slovakia, Slovenia, Sweden, Tajikistan, Turkey, Great Britain and Ukraine.

When testing for metric invariance, I released factor loading constraints on trust in the police in Sweden, Azerbaijan and Armenia, armed forces in Sweden and Britain, government in Sweden and Kosovo, and political parties in Georgia and Kosovo. The model reached partial metric invariance with good fit statistics (chi sq = 1242.068, DF = 215, RMSEA = 0.066, CFI = 0.984, SRMR = 0.058). In the scalar invariant model, I released equality constraints on intercepts on trust in the police, government and armed forces in all countries, which resulted in acceptable partial scalar invariance (chi sq = 1886.738, DF = 243, RMSEA = 0.078, CFI = 0.974, SRMR = 0.062). As a result, it is possible to compare the correlates and means of political trust across 29 countries using this latent factor (Table 2). The unstandardized factor loadings on the partial scalar invariant model are available in Table 5.

**Table 2:** Model 2: error correlation between armed forces and police (29 countries) Data source EBRD and World Bank. All available data are used in ML estimation

	Chi square	DF	RMSEA	CFI	SRMR
Configural	692.945	116	0.067	0.991	0.020
Metric	1658.009	224	0.076	0.977	0.076
Partial metric	1242.068	215	0.066	0.984	0.058
Scalar	7821.219	327	0.144	0.880	0.167
Partial scalar	1886.738	243	0.078	0.974	0.062
*Compare: bi*-*dimensional model (armed forces and police reflect separate factor, 29 countries)*					
Configural	692.945	116	0.067	0.991	0.020
Metric	1227.474	200	0.068	0.984	0.048
Scalar	4735.337	284	0.119	0.929	0.085
Partial scalar	3967.392	256	0.115	0.941	0.079

*DF* degrees of freedom, *RMSEA* root mean square error of approximation, *CFI* comparative fit index, *SRMR* standardized root mean square residual

**Fig. 3:**
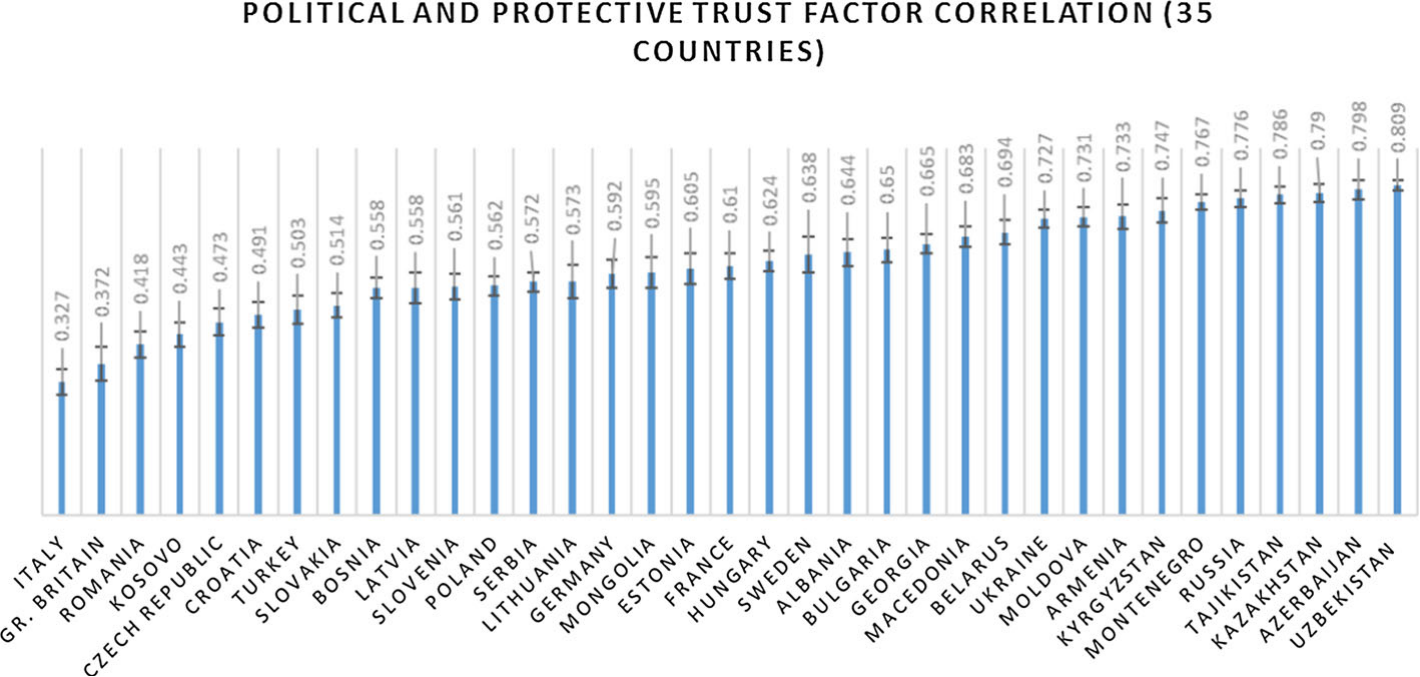
Countries are ranked by the ascending factor correlation between a political trust factor (measured by trust in the government, parliament and political parties) and a protective trust factor (measured by trust in the armed forces and police)

A bi-dimensional model in which trust in the armed forces and police is reflected by a separate factor reaches metric invariance with better fit statistics than the unidimensional model, but it does not quite reach partial scalar invariance in the same countries (chi sq = 3967.392, DF = 256, RMSEA = 0.115, CFI = 0.941, SRMR = 0.079) because I was unable to release equality constraints on a factor with two indicators. As a result, one can use the bi-dimensional model to compare correlates, but not means across countries. If we line up the countries in order of the factor correlation in this bi-dimensional model, we can visualize some of the regional variation in this measurement model. In Fig. 3, we can see that Italy has the weakest factor correlation (0.327) and Uzbekistan the largest (0.809).

Again, there appears to be clustering by regime type. The weakest factor correlations appear in Eastern and Western Europe and the strongest in the former Soviet states. This means that trust in the police and armed forces has relatively little in common with trust in political institutions in the European part of the sample, and quite a lot in the former Soviet states. Importantly, factor correlations on the former Soviet side inching close to 0.80 show that there is a lack of discriminant validity between the factors. That is, trust in the police and armed forces are appropriate measures of political trust in these countries. Specifying an error correlation or two-factor model was likely part of the reason countries on this tail of the sample had poor fit statistics and had to be excluded from invariance testing in the first place.

## Results: Model 3

In Model 3, trust in the government, parliament, political parties, courts and police load on one factor with an error correlation between the latter two indicators. Here, I test whether respondents conceive “order” or “neutral” institutions to be separate from political institutions. As in the previous models, the model would not reach configural invariance until I removed the countries with the most problematic misspecifications in JRule, where significant MIs recommend error correlations between trust in courts and political parties and between trust in parliament and government in Belarus, Bulgaria, Kazakhstan, Kosovo, Uzbekistan and Macedonia. The model still did not achieve configural invariance after removing these countries so I chose to remove countries with the next highest misspecifications in JRule suggesting complicated error correlations (Turkey, Bosnia, Azerbaijan, Moldova, Czech Republic and Ukraine). Unfortunately, the bulk of the excluded countries are former Soviet autocracies, making this model less comparable across regime types.

The amount of misspecification confirms the inconsistent loadings of trust in the courts in the initial EFA solutions: in most former Soviet states, parliaments and courts appear to be under the sway of central political institutions. This also appears in the standardized factor loadings (available in the Online Appendix). The five countries with the highest loadings for trust in courts are Azerbaijan (0.863), Armenia (0.851), Tajikistan (0.840), Montenegro (0.832) and Kosovo (0.780) while the ones with the lowest loadings are Sweden (0.442), Great Britain (0.446), Latvia (0.463), Italy (0.501) and Lithuania (0.528). Almost exactly the same pattern can be found regarding loadings for trust in the police. The five countries with the highest loadings are Azerbaijan (0.722), Montenegro (0.677), Uzbekistan (0.649), Tajikistan (0.646) and Kazakhstan (0.641) while the countries with the lowest loadings are Italy (0.290), Great Britain (0.309), Sweden (0.343), Lithuania (0.355) and Latvia (0.355). Respondents in former Soviet states and autocracies tend to associate courts and police with political institutions whereas respondents in Western Europe and the Baltics do not.

With 23 countries, the model reached acceptable fit statistics for configural invariance (chi sq = 680.399, DF = 92, RMSEA = 0.077, CFI = 0.987, SRMR = 0.020). The final 23 countries are Albania, Armenia, Croatia, Estonia, France, Georgia, Germany, Hungary, Italy, Kyrgyzstan, Latvia, Lithuania, Mongolia, Montenegro, Poland, Romania, Russia, Serbia, Slovakia, Slovenia, Sweden, Tajikistan and Great Britain.

The model did not reach metric invariance, so I used JRule to identify the most problematic items. After releasing factor loading constraints on trust in the government in Kyrgyzstan and Sweden as well as trust in the police and courts in all countries, the model reached partial metric invariance (chi sq = 915.343, DF = 134, RMSEA = 0.073, CFI = 0.983, SRMR = 0.042). The scalar invariance test produced very poor fit statistics. Although I released intercept equality constraints on trust in the police, government and political parties for all countries, the model failed to meet partial scalar invariance (chi sq = 2491.689, DF = 156, RMSEA = 0.117, CFI = 0.950, SRMR = 0.112). The bi-dimensional model in which trust in the courts and police are reflected by a separate factor performed better on metric invariance than the unidimensional model but also failed to meet partial scalar invariance.

On this basis we can use both models to compare the correlates of political trust across 23 countries, but not means (Table 3). The size of the error correlation across countries does not show clear geographical patterns, although, as expected, many former Soviet autocracies have to be excluded from invariance testing because the courts and police tend to load on federal-level political institutions. Interestingly, although this model has the most theoretical support in the literature for representing “order” or “neutral” institutions, it produces the poorest fit to the data within and across countries out of the four models mainly because the courts and police are *not* neutral or independent of political sway in a good part of the sample, contributing to disorder and undermining the rule of law rather than the reverse.

**Table 3:** Model 3: error correlation between courts and police (23 countries) Data source EBRD and World Bank. All available data are used in ML estimation

	Chi square	DF	RMSEA	CFI	SRMR
Configural	680.399	92	0.077	0.987	0.020
Metric	1580.448	180	0.084	0.970	0.082
Partial metric	915.343	134	0.073	0.983	0.042
Scalar	4216.209	222	0.128	0.915	0.234
Partial scalar	2491.689	156	0.117	0.950	0.112
*Compare: bi*-*dimensional model (courts and police reflect separate factor, 23 countries)*					
Configural	680.397	92	0.077	0.987	0.020
Metric	1086.635	158	0.073	0.980	0.051
Scalar	3680.730	224	0.119	0.926	0.087
Partial scalar	3028.274	202	0.113	0.940	0.078

*DF* degrees of freedom, *RMSEA* root mean square error of approximation, *CFI* comparative fit index, *SRMR* standardized root mean square residual

## Results: Model 4

In Model 4, trust indicators in the government, parliament, political parties, and local government load on a single ‘political trust’ factor. This specification achieved configural invariance for all 35 countries (chi sq = 453.666, DF = 70, RMSEA = 0.071, CFI = 0.994, SRMR = 0.013). Model fit deteriorated under the test for metric invariance, but after I released the problematic factor loading constraint on trust in the local government, the model achieved partial metric invariance (chi sq = 905.601, DF = 138, RMSEA = 0.072, CFI = 0.989, SRMR = 0.048). When testing for scalar invariance, I freed intercept equality constraints on trust in the local government and political parties in all countries, but the model barely failed to meet partial scalar invariance (chi sq = 1777.389, DF = 172, RMSEA = 0.093, CFI = 0.976, SRMR = 0.058). Though this can be considered a borderline case, JRule shows that significant misspecifications remain in nine countries. Any mean comparisons using this latent factor should be treated with caution (Table 4).

**Table 4:** Model 4: Simple Model. Trust in government, parliament, political parties and local government load on a single ‘political trust’ factor. (35 countries) Data source EBRD and World Bank. All available data are used in ML estimation

	Chi square	DF	RMSEA	CFI	SRMR
Configural	453.666	70	0.071	0.994	0.013
Metric	1522.867	172	0.085	0.980	0.076
Partial metric	905.601	138	0.072	0.989	0.048
Scalar	3973.355	240	0.120	0.945	0.090
Partial scalar	1777.389	172	0.093	0.976	0.058
*Compare: Simple model without trust in local government, 35 countries*					
Configural	0.001	0	0.000	1.000	0.000
Metric	432.608	68	0.070	0.992	0.052
Scalar	2274.023	136	0.120	0.953	0.075
Partial scalar	1293.938	102	0.104	0.974	0.058

*DF* degrees of freedom, *RMSEA* root mean square error of approximation, *CFI* comparative fit index, *SRMR* standardized root mean square residual

A comparative model with only three indicators (excluding trust in local government) also failed to meet partial scalar invariance (chi sq = 1293.938, DF = 102, RMSEA = 0.104, CFI = 0.974, SRMR = 0.058). As expected, a simple model of political trust without multidimensional indicators was able to reach partial metric invariance across all countries, proving the most conducive of all models to a cross-national pooled regression analysis using this survey. Comparing means of political trust using either of these simple models, however, may be problematic due to the lack of partial scalar invariance. Invariant unstandardized factor loadings for this model are available in Table 5.

**Table 5:** Unstandardized factor loadings on partial metric and scalar invariant models

Item	Model 1 Partial scalar 21 countries	Model 2 Partial scalar 29 countries	Model 3 Partial metric 23 countries	Model 4 Partial metric 35 countries
Trust in government	1.000 (0.000)	1.000 (0.000)	1.000 (0.000)	1.000 (0.000)
Trust in parliament	0.981 (0.006)	1.025 (0.007)	1.063 (0.008)	1.024 (0.006)
Trust in political parties	0.774 (0.007)	0.780 (0.006)	0.803 (0.008)	0.781 (0.006)
Trust in local gov.	0.848 (0.007)			0.745 (0.036)
Trust in regional gov.	0.898 (0.006)			
Trust in courts			0.831 (0.034)	
Trust in police		0.566 (0.007)	0.514 (0.040)	
Trust in armed forces		0.525 (0.007)		

All available data are used in ML estimation

Loadings are all significant (*p* < 0.01)

Because the four-indicator simple model managed to reach partial metric invariance for all 35 countries in the sample, I have also included unstandardized factor loadings per country for this model in Table 6. A perusal of these results helps illustrate why trust in the local government proved to be the most problematic indicator during invariance testing. The five countries with the highest loadings for trust in local government are Tajikistan (1.231), Kyrgyzstan (1.164), Azerbaijan (1.127), Uzbekistan (1.054) and Russia (1.031), while the countries with the lowest are France (0.511), Estonia (0.512), the Czech Republic (0.605), Latvia (0.628), Slovenia (0.640), Slovakia (0.650) and Lithuania (0.672). Unsurprisingly, we see that respondents from countries in Central Europe and the Baltics distinguish between local and federal levels of government, while those in former Soviet autocracies do not. This is consistent with the results in Models 1 and 3.

**Table 6:** Unstandardized factor loadings per country, Model 4

Country	Trust in government	Trust in parliament	Trust in political parties	Trust in local government
Albania	1.000 (0.000)	1.091 (0.046)	0.819 (0.041)	0.770 (0.041)
Armenia	1.000 (0.000)	1.002 (0.027)	0.791 (0.031)	0.887 (0.032)
Azerbaijan	1.000 (0.000)	1.168 (0.038)	0.948 (0.038)	1.127 (0.045)
Belarus	1.000 (0.000)	0.957 (0.026)	0.606 (0.036)	0.954 (0.028)
Bosnia	1.000 (0.000)	1.035 (0.022)	0.784 (0.026)	0.994 (0.025)
Bulgaria	1.000 (0.000)	0.978 (0.040)	0.802 (0.037)	0.824 (0.046)
Croatia	1.000 (0.000)	1.030 (0.034)	0.809 (0.033)	0.849 (0.042)
Czech Rep	1.000 (0.000)	1.072 (0.041)	0.817 (0.036)	0.605 (0.042)
Estonia	1.000 (0.000)	1.231 (0.084)	0.643 (0.052)	0.512 (0.050)
France	1.000 (0.000)	1.009 (0.059)	0.660 (0.042)	0.511 (0.047)
Georgia	1.000 (0.000)	1.005 (0.024)	0.567 (0.032)	0.973 (0.026)
Germany	1.000 (0.000)	1.002 (0.033)	0.703 (0.032)	0.791 (0.031)
Gr. Britain	1.000 (0.000)	1.021 (0.030)	0.711 (0.026)	0.783 (0.030)
Hungary	1.000 (0.000)	0.963 (0.031)	0.673 (0.028)	0.774 (0.032)
Italy	1.000 (0.000)	1.005 (0.033)	0.826 (0.032)	0.736 (0.039)
Kazakhstan	1.000 (0.000)	0.951 (0.026)	0.759 (0.032)	0.863 (0.027)
Kosovo	1.000 (0.000)	1.172 (0.039)	1.054 (0.041)	0.976 (0.037)
Kyrgyzstan	1.000 (0.000)	1.131 (0.078)	0.966 (0.073)	1.164 (0.072)
Latvia	1.000 (0.000)	1.043 (0.055)	0.718 (0.044)	0.628 (0.053)
Lithuania	1.000 (0.000)	0.993 (0.052)	0.755 (0.045)	0.672 (0.050)
Macedonia	1.000 (0.000)	0.993 (0.037)	0.750 (0.035)	0.937 (0.038)
Moldova	1.000 (0.000)	0.969 (0.022)	0.815 (0.026)	0.774 (0.028)
Mongolia	1.000 (0.000)	1.035 (0.050)	0.868 (0.047)	0.833 (0.047)
Montenegro	1.000 (0.000)	0.862 (0.022)	0.655 (0.027)	0.854 (0.024)
Poland	1.000 (0.000)	1.056 (0.030)	0.814 (0.029)	0.831 (0.028)
Romania	1.000 (0.000)	1.142 (0.046)	0.757 (0.040)	0.912 (0.055)
Russia	1.000 (0.000)	1.126 (0.036)	0.819 (0.033)	1.031 (0.036)
Serbia	1.000 (0.000)	1.062 (0.028)	0.804 (0.026)	0.892 (0.031)
Slovakia	1.000 (0.000)	1.021 (0.034)	0.838 (0.034)	0.650 (0.036)
Slovenia	1.000 (0.000)	1.039 (.046)	0.739 (.041)	0.640 (.042)
Sweden	1.000 (0.000)	0.901 (0.049)	0.698 (0.043)	0.780 (0.045)
Tajikistan	1.000 (0.000)	1.243 (0.044)	1.067 (0.055)	1.231 (0.045)
Turkey	1.000 (0.000)	0.963 (0.039)	0.640 (0.040)	0.866 (0.036)
Ukraine	1.000 (0.000)	0.969 (0.027)	0.737 (0.026)	0.764 (0.030)
Uzbekistan	1.000 (0.000)	1.050 (0.019)	0.998 (0.032)	1.054 (0.021)

All loadings are significant (*p* < 0.01)

## Conclusion

Despite growth in comparative political trust research in the last two decades, scholars have paid insufficient attention to the measurement validity and cross-national equivalence of the concept. The most common approach to measurement has consisted of taking averages or sum scores of diverse sets of indicators without theoretical justification. In this paper I showed that this common “kitchen sink” approach to measurement is inappropriate by investigating the measurement equivalence of political trust across thirty-five countries in Europe and the former Soviet space using the 2010 Life in Transition II Survey. Although issues in cross-national measurement are gaining attention in political trust research, this is the first study to examine the measurement validity and equivalence of political trust across diverse regime types.

I tested four models of political trust, finding that trust perceptions in political institutions like the government, parliament and political parties tend to differ from (1) trust in regional and local political institutions, (2) trust in protective institutions like the armed forces and police and (3) trust in order institutions like the courts and police. Measurement models with error correlations along these dimensions of political trust all reached at least partial metric invariance across *most* countries in the survey. This means that respondents in diverse cultures and regime types understand subsets of survey questions similarly, which allows us to compare the correlates of the latent factors from each model without losing substantive meaning. Coefficient estimates in a pooled regression analysis will not suffer from measurement-induced bias if these models of political trust are specified correctly and used within the structural equations framework to control for measurement error. It will not be enough to use sum scores or averages of each model’s combination of indicators.

While this outcome allows us a fair degree of optimism about the comparability of the measurement models, the only model which was comparable across all thirty-five countries was based on just four indicators (trust in the government, parliament, political parties and local government). A handful of countries, usually former Soviet autocracies, had to be excluded from invariance testing in the other models. Some variation in error correlations proved to be non-trivial across regime types. I found that trust in local and federal-level political institutions has relatively little in common in Eastern Europe, mildly more in Western Europe, and significantly more in the former Soviet space. Political trust also tends to be unrelated to trust in the police and armed forces across Europe, but strongly related in the former Soviet space, suggesting the effects of corruption and stronger central controls over these institutions. Likewise, citizens of former Soviet countries do not always perceive courts to be independent of political influence.

Two models achieved partial scalar invariance, allowing for mean comparisons on the latent factor across subsets of countries. The thirty-five country simple model of political trust, however, barely failed to reach partial scalar invariance according to conventional fit statistics. While this may appear disappointing for the prospects of cross-regime mean comparison, some advances in latent variable modeling provide reason for optimism. Oberski (2014) shows that a lack of invariance might not necessarily invalidate group comparisons and introduces the EPC-interest statistic to assess the substantive relevance of invariance misspecifications. A number of studies discuss the possibility of Bayesian techniques to establish approximate measurement invariance where traditional fit statistics appear to be overly strict (Muthén and Asparouhov 2012; Davidov et al. 2015; van de Schoot et al. 2013; Zercher et al. 2015). Using these tools in cross-regime surveys to improve invariance testing can be a fruitful direction for future research.

Substantively, this study opens interesting questions about what political trust truly means. While factor analysis illuminates the regional clustering of measurement patterns using typical survey questions, it cannot determine the nature and content of the studied beliefs, nor the precise reasons for misfit. Qualitative probing studies can be useful to capture local knowledge about political trustworthiness and to construct more cross-nationally comparable survey items. Meanwhile, it is important to improve the way we use even the simplest trust in government survey indicators in cross-national research. Although political trust is believed to have profound consequences for how we are governed and relate to each other, we cannot properly assess its causes and effects without diligently accounting for its measurement validity in diverse institutional contexts.

## Electronic supplementary material

Below is the link to the electronic supplementary material.

Supplementary material 1 (DOC 1243 kb)

## Footnotes

1 Here is the full list of countries with each sample size in parentheses: Albania (1029), Armenia (948), Azerbaijan (988), Belarus (895), Bosnia (1075), Bulgaria (1007), Croatia (997), Czech Republic (1006), Estonia (989), France (1008), Georgia (959), Germany (1032), Great Britain (1447), Hungary (1028), Italy (1046), Kazakhstan (943), Kyrgyzstan (992), Latvia (995), Lithuania (1003), Macedonia (1058), Moldova (1023), Mongolia (980), Poland (1587), Romania (1068), Russia (1549), Serbia (1506), Slovakia (995), Slovenia (984), Sweden (899), Tajikistan (996), Turkey (996), Ukraine (1547), Uzbekistan (1417), Kosovo (1081), Montenegro (966).

2 See the full methodological report on the EBRD website: http://www.ebrd.com/news/publications/special-reports/life-in-transition-survey-ii.html.

3 The EFA solutions are available in the Online Appendix.

4 I set EPC values higher than 0.15 for unstandardized factor loadings and 0.20 for intercepts as cutoff criteria to assess metric and scalar invariance output. I specify the power of the test as 0.80 and a type I error of 0.05.
