# Supplementary material for: Can We Trust Measures of Political Trust? Assessing Measurement Equivalence in Diverse Regime Types
Source: Soc Indic Res. 2016 Jul 4;133(3):963–84. doi: 10.1007/s11205-016-1400-8 (PMC5579303; doi:10.1007/s11205-016-1400-8)
Supplement: Supplementary file 1 — Supplementary material 1 (DOC 1243 kb) [file 11205_2016_1400_MOESM1_ESM.doc]

Contents:

1) Fit Statistics for Models 1-4 (p. 1-5)

2) Standardized factor loadings for each model (p. 5-9)

3) Table of trust means per country (p. 9-10)

4) EFA output per country (p. 11-28)

5) Mplus input code for CFA (p.28)

1. Global Fit Statistics for each model

Fit statistics: Model 1 Regional and Local Political Trust

| Country | Chi Square | RMSEA | CFI | SRMR | Reg & Loc Error  Corr (SE) | Modification Index (EPC),  standardized |
| --- | --- | --- | --- | --- | --- | --- |
| Azerbaijan | 27.055 | 0.076 | 0.993 | 0.013 | .199 (.047) |  |
| Turkey | 54.334 | 0.112 | 0.979 | 0.023 | .252 (.043) | REG WITH GOV 42.165 (.344) |
| Tajikistan | 116.876 | 0.168 | 0.966 | 0.026 | .298 (.055) | REG WITH GOV 112.105 (.644)  LOC WITH GOV 60.538 (-.370) |
| Belarus | 55.132 | 0.12 | 0.986 | 0.015 | .321 (.046) | PARL WITH REG 46.304 (-.421)  REG WITH GOV 46.037 (.712) |
| Uzbekistan | 63.597 | 0.103 | 0.991 | 0.012 | .323 (.035) | PARL WITH REG 31.897 (-.266)  REG WITH GOV 31.484 (.273) |
| Bosnia | 92.748 | 0.144 | 0.983 | 0.015 | .329 (.038) | REG WITH GOV 83.420 (.512)  PARL WITH REG 40.389 (-.363) |
| Ukraine | 42.524 | 0.079 | 0.991 | 0.013 | .388 (.027) | REG WITH GOV 32.756 (.277) |
| Russia | 96.368 | 0.122 | 0.979 | 0.021 | .422 (.029) | REG WITH GOV 79.976 (.337) |
| Kyrgyzstan | 117.887 | 0.169 | 0.945 | 0.045 | .435 (.045) | POLP WITH PARL 98.104 (.422)  REG WITH GOV 65.924 (.417) |
| France | 10.609 | 0.04 | 0.995 | 0.012 | .447 (.027) |  |
| Estonia | 25.617 | 0.074 | 0.98 | 0.023 | .455 (.033) |  |
| Sweden | 6.946 | 0.029 | 0.998 | 0.009 | .455 (.034) |  |
| Serbia | 73.822 | 0.108 | 0.984 | 0.015 | .478 (.025) | REG WITH GOV 71.166 (.303)  LOC WITH GOV 42.352 (-0.201) |
| Germany | 21.836 | 0.066 | 0.994 | 0.013 | .495 (.028) |  |
| Czech Republic | 21.142 | 0.065 | 0.992 | 0.018 | .515 (.025) |  |
| Kazakhstan | 22.489 | 0.07 | 0.995 | 0.011 | .516 (.030) |  |
| Armenia | 45.584 | 0.105 | 0.988 | 0.015 | .522 (.029) | POLP WITH PARL 33.648 (.282)  REG WITH GOV 31.635 (.319) |
| Slovakia | 13.838 | 0.05 | 0.996 | 0.01 | .529 (.026) |  |
| Italy | 30.85 | 0.08 | 0.991 | 0.013 | .531 (.024) | REG WITH GOV 31.029 (.230) |
| Moldova | 30.485 | 0.08 | 0.993 | 0.009 | .557 (.026) |  |
| Mongolia | 29.031 | 0.080 | 0.988 | 0.019 | .569 (.028) |  |
| Lithuania | 12.378 | 0.046 | 0.995 | 0.015 | .580 (.026) |  |
| Georgia | 15.397 | 0.055 | 0.997 | 0.011 | .594 (.028) |  |
| Bulgaria | 57.393 | 0.115 | 0.978 | 0.026 | .595 (.025) | POLP WITH PARL 36.549 (.391)  POLP WITH GOV 32.086 (-.313) |
| Albania | 113.962 | 0.163 | 0.957 | 0.029 | .598 (.023) | REG WITH GOV 91.411 (.449)  PARL WITH REG 70.629 (-.296) |
| Poland | 72.349 | 0.104 | 0.986 | 0.019 | .638 (.017) | POLP WITH PARL 51.173 (.317)  REG WITH GOV 45.010 (.180) |
| Romania | 43.027 | 0.096 | 0.984 | 0.017 | .665 (.018) | REG WITH GOV 30.630 (.174) |
| Croatia | 26.129 | 0.074 | 0.993 | 0.012 | .707 (.017) |  |

Note:

All correlations are significant (p<0.01)

REG= regional government; LOC=local government, PARL=parliament, GOV=government, POLP=political parties. WITH = suggestion of an error correlation.

Fit Statistics: Model 2 Political and Protective Trust (Bi-dimensional model)

| Country | Chi Square | RMSEA | CFI | SRMR | Factor Corr | Modification Index (EPC),  standardized |
| --- | --- | --- | --- | --- | --- | --- |
| Italy | 9.44 | 0.036 | 0.998 | 0.014 | 0.327 (.032) |  |
| Great Britain | 3.519 | 0 | 1 | 0.006 | 0.372 (.040) |  |
| Romania | 6.375 | 0.024 | 0.999 | 0.014 | 0.418 (.032) |  |
| Kosovo | 26.81 | 0.073 | 0.991 | 0.013 | 0.443 (.030) |  |
| Czech Rep | 24.379 | 0.071 | 0.990 | 0.024 | 0.473 (.033) |  |
| Croatia | 10.933 | 0.042 | 0.997 | 0.012 | 0.491 (.031) |  |
| Turkey | 11.895 | 0.044 | 0.995 | 0.015 | 0.503 (.034) |  |
| Slovakia | 22.731 | 0.068 | 0.991 | 0.02 | 0.514 (.030) |  |
| Bosnia | 90.723 | 0.142 | 0.974 | 0.042 | 0.558 (.025) | PROTECT BY POLP 50.043 (.206)  PARL WITH GOV 50.038 (2.011) |
| Latvia | 17.92 | 0.059 | 0.988 | 0.017 | 0.558 (.038) |  |
| Slovenia | 35.172 | 0.089 | 0.981 | 0.024 | 0.561 (.032) |  |
| Poland | 17.315 | 0.046 | 0.996 | 0.012 | 0.562 (.023) |  |
| Serbia | 8.506 | 0.027 | 0.999 | 0.008 | 0.572 (.023) |  |
| Lithuania | 18.094 | 0.059 | 0.988 | 0.023 | 0.573 (.040) |  |
| Germany | 16.202 | 0.054 | 0.993 | 0.017 | 0.592 (.035) |  |
| Mongolia | 9.704 | 0.038 | 0.995 | 0.013 | 0.595 (.037) |  |
| Estonia | 26.477 | 0.075 | 0.978 | 0.024 | 0.605 (.038) |  |
| France | 3.479 | 0 | 1 | 0.009 | 0.610 (.031) |  |
| Hungary | 42.114 | 0.096 | 0.985 | 0.032 | 0.624 (.025) | PROTECT BY POLP 40.678 (.237)  PARL WITH GOV 40.690 (983) |
| Sweden | 14.107 | 0.053 | 0.988 | 0.021 | 0.638 (.044) |  |
| Albania | 14.291 | 0.05 | 0.993 | 0.018 | 0.644 (.032) |  |
| Bulgaria | 28.084 | 0.078 | 0.987 | 0.022 | 0.650 (.029) |  |
| Georgia | 6.591 | 0.026 | 0.999 | 0.013 | 0.665 (.024) |  |
| Macedonia | 18.142 | 0.058 | 0.994 | 0.016 | 0.683 (.024) |  |
| Belarus | 52.419 | 0.114 | 0.973 | 0.037 | 0.694 (.029) | POL WITH POLP 45.759 (.389)  PROTECT BY POLP 36.044 (.331) |
| Ukraine | 33.387 | 0.069 | 0.990 | 0.021 | 0.727 (.023) | PROTECT BY POLP 31.092 (.250)  PARL WITH GOV 31.078 (.626) |
| Moldova | 45.722 | 0.101 | 0.986 | 0.027 | 0.731 (.024) | PROTECT BY POLP 37.570 (.267)  PARL WITH GOV 37.544 (1.170) |
| Armenia | 20.414 | 0.065 | 0.992 | 0.015 | 0.733 (.030) |  |
| Kyrgyzstan | 23.286 | 0.07 | 0.984 | 0.022 | 0.747 (.030) |  |
| Montenegroo | 46.607 | 0.105 | 0.985 | 0.027 | 0.767 (.019) | PROTECT BY POLP 24.899 (.245)  PARL WITH GOV 24.886 (.841) |
| Russia | 18.488 | 0.048 | 0.995 | 0.012 | 0.776 (.021) |  |
| Tajikistan | 43.479 | 0.099 | 0.982 | 0.026 | 0.786 (.021) | PROTECT BY PARL 28.542 (-.422)  POLP WITH GOV 28.536 (-.275) |
| Kazakhstan | 65.451 | 0.127 | 0.973 | 0.036 | 0.790 (.023) | PROTECT BY POLP 64.943 (.501)  PROTECT WITH GOV 64.942 (1.233) |
| Azerbaijan | 56.406 | 0.115 | 0.977 | 0.028 | 0.798 (.025) | POLP WITH GOV 43.184 (.519)  PROTECT BY GOV 43.180 (.382) |
| Uzbekistan | 178.838 | 0.175 | 0.961 | 0.046 | 0.809 (.014) | PROTECT BY POLP 121.964 (.510)  PARL WITH GOV 121.952 (1.686) |

Note:

All correlations are significant (p<0.01)

PARL=parliament, GOV=government, POLP=political parties, PROTECT = protective institutional trust factor, POLIT = political trust factor. WITH = suggestion of an error correlation. BY = suggestion of an indicator loading on the specified factor.

Fit Statistics: Model 3 Courts and Police Error Correlation

| Country | Chi Square | RMSEA | CFI | SRMR | Error Corr | Modification Index (EPC),  standardized |
| --- | --- | --- | --- | --- | --- | --- |
| Kosovo | 42.680 | 0.094 | 0.984 | 0.019 | -0.050 (.035) | COURTS WITH POLP 38.429 |
| Albania | 46.761 | 0.102 | 0.976 | 0.024 | 0.056 (.036) | COURTS WITH GOV 33.882 (-.271) |
| Georgia | 91.029 | 0.149 | 0.964 | 0.036 | 0.089 (.039) | COURTS WITH POLP 62.319 (.309) |
| Mongolia | 13.936 | 0.050 | 0.992 | 0.015 | 0.104 (.037) |  |
| Tajikistan | 54.436 | 0.112 | 0.978 | 0.026 | 0.135 (.045) |  |
| Azerbaijan | 53.178 | 0.111 | 0.984 | 0.021 | 0.149 (.042) | POLICE WITH GOV 38.830 (.241) |
| Macedonia | 45.438 | 0.099 | 0.980 | 0.021 | 0.154 (.035) | POLICE WITH GOV 37.244 |
| Montenegro | 48.297 | 0.107 | 0.986 | 0.019 | 0.198 (.037) | PARL WITH GOV 32.852 (.640) |
| Estonia | 19.052 | 0.062 | 0.985 | 0.021 | 0.204 (.037) |  |
| Italy | 51.661 | 0.107 | 0.875 | 0.025 | 0.209 (.031) | COURTS WITH POLP 34.431 (.203) |
| Bulgaria | 80.042 | 0.138 | 0.959 | 0.034 | 0.236 (.035) | PARL WITH GOV 47.837 (.544) |
| France | 26.188 | 0.074 | 0.983 | 0.018 | 0.242 (.033) |  |
| Croatia | 20.695 | 0.065 | 0.992 | 0.015 | 0.251 (.032) |  |
| Latvia | 6.581 | 0.025 | 0.998 | 0.012 | 0.266 (.034) |  |
| Britain | 7.865 | 0.026 | 0.998 | 0.009 | 0.280 (.025) |  |
| Turkey | 25.545 | 0.073 | 0.986 | 0.021 | 0.280 (.033) |  |
| Romania | 22.427 | 0.066 | 0.990 | 0.018 | 0.284 (.031) |  |
| Sweden | 18.443 | 0.063 | 0.984 | 0.023 | 0.284 (.033) |  |
| Belarus | 65.173 | 0.128 | 0.970 | 0.043 | 0.285 (.034) |  |
| Armenia | 26.308 | 0.076 | 0.992 | 0.017 | 0.291 (.039) | PARL WITH GOV 56.504 (1.856) |
| Serbia | 12.496 | 0.038 | 0.997 | 0.009 | 0.295 (.026) |  |
| Kyrgyzstan | 23.271 | 0.069 | 0.984 | 0.021 | 0.298 (.036) |  |
| Kazakhstan | 102.756 | 0.161 | 0.963 | 0.038 | 0.351 (.034) | PARL WITH GOV 105.577 (1.085) |
| Lithuania | 21.023 | 0.065 | 0.987 | 0.024 | 0.356 (.030) |  |
| Czech Rep | 61.840 | 0.120 | 0.971 | 0.032 | 0.375 (.029) | PARL WITH GOV 58.853 (.899) |
| Poland | 26.718 | 0.060 | 0.993 | 0.013 | 0.376 (.024) |  |
| Russia | 28.776 | 0.063 | 0.992 | 0.017 | 0.380 (.027) |  |
| Slovakia | 31.169 | 0.082 | 0.988 | 0.022 | 0.395 (.029) |  |
| Moldova | 59.483 | 0.116 | 0.983 | 0.028 | 0.407 (.030) | PARL WITH GOV 41.269 |
| Hungary | 42.901 | 0.097 | 0.984 | 0.026 | 0.426 (.028) |  |
| Ukraine | 40.486 | 0.077 | 0.989 | 0.023 | 0.429 (.023) | PARL WITH GOV 34.879 |
| Bosnia | 100.788 | 0.150 | 0.971 | 0.035 | 0.432 (.026) | PARL WITH GOV 91.344 (1.721) |
| Germany | 8.843 | 0.034 | 0.998 | 0.013 | 0.440 (.026) |  |
| Uzbekistan | 236.213 | 0.202 | 0.951 | 0.052 | 0.496 (.023) | PARL WITH GOV 172.738 (1.964) |

All correlations are significant (p<0.01)

PARL=parliament, GOV=government, POLP=political parties, WITH = suggestion of an error correlation.

Fit statistics: Model 4 (Simple)

| Country | Chi Square | RMSEA | CFI | SRMR | Modification Index (EPC),  standardized |
| --- | --- | --- | --- | --- | --- |
| Albania | 15.208 | 0.080 | 0.991 | 0.018 |  |
| Armenia | 9.499 | 0.063 | 0..997 | 0.010 |  |
| Azerbaijan | 7.280 | 0.051 | 0.997 | 0.010 |  |
| Belarus | 1.499 | 0.000 | 1.000 | 0.004 |  |
| Bosnia | 15.152 | 0.078 | 0.996 | 0.009 |  |
| Bulgaria | 39.545 | 0.137 | 0.975 | 0.024 | PARL WITH LOC 33.975 (-.326)  POLP WITH GOV 33.983 (-.373) |
| Croatia | 5.515 | 0.042 | 0.998 | 0.008 |  |
| Czech Rep | 13.745 | 0.076 | 0.992 | 0.017 |  |
| Estonia | 2.085 | 0.007 | 1.000 | 0.010 |  |
| France | 2.362 | 0.013 | 1.000 | 0.008 |  |
| Georgia | 3.063 | 0.024 | 1.000 | 0.007 |  |
| Germany | 15.686 | 0.081 | 0.993 | 0.013 |  |
| Great Britain | 3.173 | 0.020 | 1.000 | 0.006 |  |
| Hungary | 24.328 | 0.104 | 0.989 | 0.020 | PARL WITH GOV 22.975 (-0.667)  POLP WITH LOC 22.967 (-0.178) |
| Italy | 0.541 | 0.000 | 1.000 | 0.003 |  |
| Kazakhstan | 9.819 | 0.064 | 0.996 | 0.009 |  |
| Kosovo | 34.128 | 0.122 | 0.986 | 0.019 | LOC WITH GOV 34.900 (0.266)  POLP WITH PARL 34.903 (0.425) |
| Kyrgyzstan | 53.980 | 0.162 | 0.946 | 0.036 | LOC WITH GOV 54.637 (.402)  POLP WITH PARL 54.602 (.467) |
| Latvia | 9.849 | 0.063 | 0.992 | 0.015 |  |
| Lithuania | 4.604 | 0.036 | 0.997 | 0.011 |  |
| Macedonia | 1.808 | 0.000 | 1.000 | 0.005 |  |
| Moldova | 7.092 | 0.050 | 0.998 | 0.006 |  |
| Mongolia | 9.581 | 0.062 | 0.994 | 0.015 |  |
| Montenegro | 47.674 | 0.154 | 0.982 | 0.018 | PARL WITH LOC 38.086 (-.404)  POLP WITH GOV 38.083 (-.115) |
| Poland | 24.925 | 0.085 | 0.992 | 0.015 | LOC WITH GOV 23.558 (.194)  POLP WITH PARL 23.558 (.266) |
| Romania | 5.542 | 0.041 | 0.997 | 0.011 |  |
| Russia | 9.817 | 0.050 | 0.997 | 0.010 |  |
| Serbia | 2.136 | 0.007 | 1.000 | 0.004 |  |
| Slovakia | 5.179 | 0.040 | 0.998 | 0.008 |  |
| Slovenia | 7.534 | 0.053 | 0.995 | 0.012 |  |
| Sweden | 2.971 | 0.023 | 0.999 | 0.008 |  |
| Tajikistan | 7.280 | 0.051 | 0.997 | 0.010 |  |
| Turkey | 13.177 | 0.075 | 0.993 | 0.015 |  |
| Ukraine | 4.193 | 0.027 | 0.999 | 0.007 |  |
| Uzbekistan | 27.013 | 0.094 | 0.994 | 0.012 |  |

Note:

PARL=parliament, GOV=government, POLP=political parties, LOC= local government, WITH = suggestion of an error correlation.

1. Standardized factor loadings for each model

STANDARDIZED FACTOR LOADINGS Model 1 Trust in political institutions in relation to trust in local and regional government

| **Country** | **Trust in Government** | **Trust in parliament** | **Trust in Political Parties** | **Trust in Local Government** | **Trust in Regional Government** |
| --- | --- | --- | --- | --- | --- |
| Azerbaijan | 0.797 (0.014) | 0.897 (0.009) | 0.766 (0.015) | 0.871 (0.011) | 0.840 (0.013) |
| Turkey | 0.817 (0.014) | 0.819 (0.015) | 0.521 (0.026) | 0.780 (0.017) | 0.803 (0.016) |
| Tajikistan | 0.840 (0.012) | 0.842 (0.013) | 0.587 (0.024) | 0.852 (0.013) | 0.908 (0.010) |
| Belarus | 0.945 (0.006) | 0.863 (0.010) | 0.549 (0.026) | 0.837 (0.013) | 0.933 (0.007) |
| Uzbekistan | 0.925 (0.005) | 0.920 (0.005) | 0.726 (0.014) | 0.883 (0.007) | 0.927 (0.005) |
| Bosnia | 0.916 (0.006) | 0.919 (0.006) | 0.723 (0.016) | 0.859 (0.009) | 0.933 (0.006) |
| Ukraine | 0.877 (0.009) | 0.841 (0.010) | 0.694 (0.015) | 0.629 (0.018) | 0.836 (0.010) |
| Russia | 0.809 (0.012) | 0.879 (0.010) | 0.668 (0.017) | 0.773 (0.013) | 0.842 (0.011) |
| Kyrgyzstan | 0.708 (0.021) | 0.667 (0.025) | 0.573 (0.028) | 0.742 (0.023) | 0.824 (0.021) |
| France | 0.735 (0.021) | 0.803 (0.020) | 0.591 (0.025) | 0.399 (0.031) | 0.548 (0.027) |
| Estonia | 0.733 (0.025) | 0.832 (0.025) | 0.475 (0.031) | 0.386 (0.032) | 0.539 (0.034) |
| Sweden | 0.785 (0.020) | 0.808 (0.021) | 0.611 (0.025) | 0.687 (0.024) | 0.687 (0.024) |
| Serbia | 0.855 (0.010) | 0.880 (0.009) | 0.722 (0.014) | 0.696 (0.015) | 0.784 (0.013) |
| Germany | 0.893 (0.010) | 0.838 (0.012) | 0.649 (0.020) | 0.708 (0.018) | 0.809 (0.013) |
| Czech Republic | 0.837 (0.014) | 0.870 (0.013) | 0.700 (0.019) | 0.468 (0.027) | 0.613 (0.023) |
| Kazakhstan | 0.918 (0.008) | 0.872 (0.010) | 0.692 (0.020) | 0.806 (0.014) | 0.857 (0.011) |
| Armenia | 0.921 (0.008) | 0.898 (0.009) | 0.723 (0.018) | 0.752 (0.016) | 0.873 (0.010) |
| Slovakia | 0.867 (0.012) | 0.860 (0.012) | 0.732 (0.017) | 0.578 (0.024) | 0.740 (0.017) |
| Italy | 0.873 (0.012) | 0.859 (0.012) | 0.721 (0.017) | 0.569 (0.023) | 0.713 (0.018) |
| Moldova | 0.933 (0.007) | 0.909 (0.008) | 0.783 (0.014) | 0.703 (0.017) | 0.843 (0.011) |
| Lithuania | 0.781 (0.020) | 0.798 (0.019) | 0.623 (0.024) | 0.480 (0.029) | 0.643 (0.025) |
| Georgia | 0.931 (0.007) | 0.906 (0.008) | 0.532 (0.025) | 0.855 (0.011) | 0.884 (0.010) |
| Bulgaria | 0.794 (0.016) | 0.836 (0.015) | 0.737 (0.019) | 0.607 (0.024) | 0.720 (0.020) |
| Mongolia | 0.815 (0.017) | 0.781 (0.019) | 0.675 (0.022) | 0.634 (0.025) | 0.741 (0.020) |
| Albania | 0.870 (0.015) | 0.785 (0.017) | 0.624 (0.023) | 0.604 (0.023) | 0.713 (0.019) |
| Poland | 0.834 (0.011) | 0.866 (0.010) | 0.696 (0.015) | 0.706 (0.015) | 0.746 (0.014) |
| Romania | 0.809 (0.016) | 0.863 (0.015) | 0.624 (0.023) | 0.534 (0.025) | 0.612 (0.023) |
| Croatia | 0.859 (0.012) | 0.866 (0.012) | 0.735 (0.017) | 0.630 (0.022) | 0.699 (0.019) |

Note: All loadings are significant at p<0.05.

STANDARDIZED FACTOR LOADINGS Model 2 Trust in political and protective institutions

| **Country** | **Trust in**  **Government** | **Trust in Parliament** | **Trust in Political Parties** | **Trust in**  **Police** | **Trust in Armed**  **Forces** |
| --- | --- | --- | --- | --- | --- |
| Italy | 0.845 (0.014) | 0.884 (0.013) | 0.728 (0.017) | 0.891 (0.032) | 0.887 (0.032) |
| Great Britain | 0.831 (0.013) | 0.866 (0.013) | 0.694 (0.016) | 0.835 (0.071) | 0.415 (0.041) |
| Romania | 0.769 (0.018) | 0.907 (0.016) | 0.615 (0.023) | 0.887 (0.032) | 0.787 (0.030) |
| Kosovo | 0.767 (0.016) | 0.919 (0.012) | 0.768 (0.016) | 0.872 (0.028) | 0.816 (0.027) |
| Czech Rep | 0.812 (0.015) | 0.883 (0.014) | 0.718 (0.019) | 0.861 (0.034) | 0.726 (0.031) |
| Croatia | 0.844 (0.014) | 0.882 (0.013) | 0.737 (0.018) | 0.827 (0.030) | 0.782 (0.029) |
| Turkey | 0.757 (0.021) | 0.882 (0.020) | 0.540 (0.026) | 0.888 (0.034) | 0.688 (0.031) |
| Slovakia | 0.848 (0.014) | 0.873 (0.013) | 0.744 (0.017) | 0.884 (0.028) | 0.754 (0.027) |
| Bosnia | 0.871 (0.010) | 0.958 (0.008) | 0.743 (0.015) | 0.925 (0.020) | 0.799 (0.020) |
| Latvia | 0.749 (0.022) | 0.849 (0.020) | 0.613 (0.025) | 0.647 (0.037) | 0.729 (0.038) |
| Slovenia | 0.786 (0.018) | 0.871 (0.017) | 0.629 (0.023) | 0.820 (0.030) | 0.726 (0.029) |
| Poland | 0.805 (0.013) | 0.888 (0.011) | 0.713 (0.015) | 0.831 (0.020) | 0.802 (0.020) |
| Serbia | 0.842 (0.011) | 0.890 (0.010) | 0.728 (0.014) | 0.896 (0.021) | 0.735 (0.020) |
| Lithuania | 0.748 (0.021) | 0.816 (0.020) | 0.648 (0.024) | 0.617 (0.038) | 0.658 (0.040) |
| Germany | 0.838 (0.015) | 0.887 (0.014) | 0.667 (0.020) | 0.708 (0.036) | 0.590 (0.034) |
| Mongolia | 0.767 (0.021) | 0.824 (0.020) | 0.698 (0.022) | 0.679 (0.035) | 0.684 (0.035) |
| Estonia | 0.760 (0.025) | 0.802 (0.025) | 0.479 (0.031) | 0.666 (0.035) | 0.693 (0.036) |
| France | 0.767 (0.022) | 0.780 (0.021) | 0.577 (0.026) | 0.802 (0.028) | 0.729 (0.028) |
| Hungary | 0.840 (0.013) | 0.893 (0.012) | 0.722 (0.018) | 0.840 (0.019) | 0.866 (0.019) |
| Sweden | 0.782 (0.023) | 0.802 (0.025) | 0.615 (0.027) | 0.528 (0.038) | 0.730 (0.044) |
| Albania | 0.797 (0.017) | 0.832 (0.016) | 0.681 (0.021) | 0.653 (0.020) | 0.741 (0.031) |
| Bulgaria | 0.755 (0.018) | 0.860 (0.015) | 0.754 (0.018) | 0.831 (0.025) | 0.749 (0.025) |
| Georgia | 0.930 (0.011) | 0.905 (0.012) | 0.538 (0.025) | 0.852 (0.019) | 0.820 (0.020) |
| Macedonia | 0.811 (0.016) | 0.834 (0.016) | 0.682 (0.020) | 0.853 (0.019) | 0.812 (0.019) |
| Belarus | 0.893 (0.012) | 0.913 (0.012) | 0.580 (0.026) | 0.867 (0.029) | 0.619 (0.029) |
| Ukraine | 0.834 (0.011) | 0.876 (0.010) | 0.710 (0.015) | 0.724 (0.021) | 0.693 (0.021) |
| Moldova | 0.906 (0.008) | 0.931 (0.008) | 0.796 (0.013) | 0.746 (0.022) | 0.780 (0.022) |
| Armenia | 0.874 (0.011) | 0.938 (0.009) | 0.745 (0.017) | 0.868 (0.030) | 0.533 (0.029) |
| Kyrgyzstan | 0.518 (0.029) | 0.752 (0.024) | 0.725 (0.024) | 0.709 (0.025) | 0.798 (0.025) |
| Montenegro | 0.893 (0.010) | 0.914 (0.009) | 0.696 (0.018) | 0.886 (0.015) | 0.809 (0.017) |
| Russia | 0.775 (0.014) | 0.889 (0.012) | 0.707 (0.017) | 0.782 (0.019) | 0.713 (0.019) |
| Tajikistan | 0.786 (0.017) | 0.858 (0.015) | 0.672 (0.022) | 0.827 (0.017) | 0.841 (0.017) |
| Kazakhstan | 0.876 (0.011) | 0.901 (0.011) | 0.730 (0.019) | 0.803 (0.021) | 0.735 (0.022) |
| Azerbaijan | 0.806 (0.015) | 0.901 (0.011) | 0.758 (0.016) | 0.908 (0.023) | 0.562 (0.026) |
| Uzbekistan | 0.905 (0.007) | 0.936 (0.006) | 0.731 (0.015) | 0.805 (0.013) | 0.886 (0.012) |

Note: All loadings are significant at p<0.05.

STANDARDIZED FACTOR LOADINGS Model 3 Courts And Police Error Correlation

| **Country** | **Trust in**  **Government** | **Trust in Parliament** | **Trust in Political Parties** | **Trust in**  **Police** | **Trust in Courts** |
| --- | --- | --- | --- | --- | --- |
| Kosovo | 0.758 (.015) | 0.903 (.010) | 0.794 (.014) | 0.385 (.028) | 0.780 (.015) |
| Albania | 0.758 (.017) | 0.863 (.014) | 0.686 (.020) | 0.413 (.029) | 0.693 (.020) |
| Georgia | 0.895 (.009) | 0.934 (.008) | 0.561 (.025) | 0.561 (.024) | 0.768 (.016) |
| Mongolia | 0.743 (.020) | 0.817 (.018) | 0.730 (.021) | 0.404 (.032) | 0.631 (.025) |
| Tajikistan | 0.756 (.017) | 0.880 (.012) | 0.681 (.020) | 0.646 (.023) | 0.840 (.014) |
| Azerbaijan | 0.786 (.014) | 0.905 (.009) | 0.776 (.015) | 0.722 (.018) | 0.863 (.011) |
| Macedonia | 0.776 (.016) | 0.866 (.013) | 0.684 (.019) | 0.571 (.024) | 0.709 (.019) |
| Montenegro | 0.877 (.009) | 0.925 (.008) | 0.705 (.018) | 0.677 (.019) | 0.832 (.012) |
| Estonia | 0.733 (.023) | 0.825 (.022) | 0.494 (.030) | 0.394 (.033) | 0.537 (.030) |
| Italy | 0.830 (.013) | 0.890 (.012) | 0.739 (.017) | 0.290 (.030) | 0.501 (.026) |
| Bulgaria | 0.734 (.018) | 0.859 (.015) | 0.774 (.018) | 0.544 (.026) | 0.598 (.025) |
| France | 0.727 (.021) | 0.813 (.019) | 0.588 (.025) | 0.484 (.029) | 0.565 (.026) |
| Croatia | 0.827 (.013) | 0.891 (.011) | 0.747 (.017) | 0.405 (.029) | 0.661 (.020) |
| Latvia | 0.718 (.022) | 0.876 (.019) | 0.615 (.025) | 0.355 (.032) | 0.463 (.031) |
| Britain | 0.822 (.013) | 0.873 (.012) | 0.695 (.016) | 0.309 (.026) | 0.446 (.023) |
| Turkey | 0.745 (.019) | 0.885 (.016) | 0.555 (.016) | 0.448 (.029) | 0.638 (.023) |
| Romania | 0.762 (.016) | 0.900 (.013) | 0.635 (.022) | 0.377 (.029) | 0.708 (.019) |
| Sweden | 0.762 (.024) | 0.816 (.025) | 0.619 (.027) | 0.343 (.035) | 0.442 (.033) |
| Belarus | 0.880 (.012) | 0.923 (.010) | 0.588 (.026) | 0.598 (.024) | 0.651 (.022) |
| Armenia | 0.867 (.010) | 0.939 (.007) | 0.754 (.017) | 0.640 (.022) | 0.851 (.012) |
| Serbia | 0.834 (.011) | 0.893 (.009) | 0.734 (.014) | 0.510 (.021) | 0.664 (.017) |
| Kyrgyzstan | 0.510 (.029) | 0.782 (.023) | 0.706 (.024) | 0.526 (.030) | 0.647 (.026) |
| Kazakhstan | 0.868 (011) | 0.893 (.010) | 0.749 (.017) | 0.641 (.022) | 0.773 (.017) |
| Lithuania | 0.743 (.021) | 0.812 (.019) | 0.656 (.024) | 0.355 (.032) | 0.528 (.028) |
| Czech Rep | 0.800 (.015) | 0.881 (.013) | 0.736 (.018) | 0.410 (.029) | 0.590 (.024) |
| Poland | 0.798 (.012) | 0.889 (.010) | 0.719 (.014) | 0.466 (.022) | 0.693 (.016) |
| Russia | 0.756 (.014) | 0.897 (.011) | 0.719 (.016) | 0.600 (.020) | 0.671 (.018) |
| Slovakia | 0.838 (.013 | 0.878 (.012) | 0.751 (.017) | 0.452 (.028) | 0.635 (.022) |
| Moldova | 0.897 (.008) | 0.938 (.007) | 0.798 (.013) | 0.544 (.024) | 0.738 (.016) |
| Hungary | 0.831 (.013) | 0.906 (.011) | 0.712 (.018) | 0.522 (.025) | 0.636 (.021) |
| Ukraine | 0.827 (.011) | 0.880 (.010) | 0.713 (.015) | 0.526 (.021) | 0.641 (.017) |
| Bosnia | 0.865 (.009) | 0.960 (.007) | 0.748 (.015) | 0.516 (0.024) | 0.701 (.017) |
| Germany | 0.833 (.014) | 0.893 (.013) | 0.664 (.020) | 0.418 (.028) | 0.561 (.024) |
| Uzbekistan | 0.897 (.007) | 0.945 (.006) | 0.734 (.015) | 0.649 (.017) | 0.748 (.013) |

STANDARDIZED FACTOR LOADINGS Simple Political Trust Model

| **Country** | **Trust in**  **Government** | **Trust in**  **Parliament** | **Trust in**  **Political**  **Parties** | **Trust in**  **Local**  **Government** |
| --- | --- | --- | --- | --- |
| Albania | 0.800 (0.017) | 0.852 (0.016) | 0.646 (0.022) | 0.611 (0.023) |
| Armenia | 0.889 (0.010) | 0.926 (0.009) | 0.736 (0.018) | 0.752 (0.016) |
| Azerbaijan | 0.790 (0.014) | 0.909 (0.009) | 0.762 (0.016) | 0.866 (0.011) |
| Belarus | 0.907 (0.009) | 0.898 (0.010) | 0.559 (0.027) | 0.853 (0.012) |
| Bosnia | 0.887 (0.008) | 0.943 (0.006) | 0.741 (0.015) | 0.856 (0.010) |
| Bulgaria | 0.774 (0.017) | 0.849 (0.015) | 0.749 (0.018) | 0.602 (0.024) |
| Croatia | 0.844 (0.013) | 0.879 (0.012) | 0.740 (0.017) | 0.626 (0.022) |
| Czech Rep | 0.822 (0.016) | 0.884 (0.014) | 0.703 (0.019) | 0.465 (0.027) |
| Estonia | 0.689 (0.027) | 0.882 (0.027) | 0.476 (0.031) | 0.382 (0.032) |
| France | 0.735 (0.023) | 0.813 (0.023) | 0.576 (0.026) | 0.399 (0.031) |
| Georgia | 0.923 (0.008) | 0.913 (0.008) | 0.538 (0.025) | 0.857 (0.011) |
| Germany | 0.879 (0.012) | 0.851 (0.013) | 0.654 (0.020) | 0.707 (0.018) |
| Great Britain | 0.832 (0.012) | 0.861 (0.011) | 0.699 (0.016) | 0.670 (0.017) |
| Hungary | 0.851 (0.013) | 0.903 (0.011) | 0.685 (0.019) | 0.695 (0.018) |
| Italy | 0.848 (0.013) | 0.880 (0.012) | 0.730 (0.017) | 0.569 (0.023) |
| Kazakhstan | 0.907 (0.010) | 0.884 (0.010) | 0.697 (0.020) | 0.809 (0.014) |
| Kosovo | 0.796 (0.014) | 0.898 (0.011) | 0.763 (0.015) | 0.754 (0.016) |
| Kyrgyzstan | 0.622 (0.027) | 0.748 (0.024) | 0.635 (0.027) | 0.708 (0.025) |
| Latvia | 0.743 (0.022) | 0.851 (0.020) | 0.619 (0.025) | 0.430 (0.031) |
| Lithuania | 0.760 (0.021) | 0.820 (0.020) | 0.625 (0.025) | 0.479 (0.029) |
| Macedonia | 0.777 (0.016) | 0.870 (0.013) | 0.680 (0.019) | 0.763 (0.016) |
| Moldova | 0.917 (0.008) | 0.927 (0.008) | 0.785 (0.014) | 0.704 (0.017) |
| Mongolia | 0.782 (0.020) | 0.814 (0.019) | 0.684 (0.023) | 0.646 (0.024) |
| Montenegro | 0.934 (0.008) | 0.880 (0.010) | 0.669 (0.020) | 0.817 (0.012) |
| Poland | 0.812 (0.012) | 0.887 (0.010) | 0.701 (0.015) | 0.702 (0.015) |
| Romania | 0.782 (0.017) | 0.892 (0.015) | 0.620 (0.023) | 0.526 (0.025) |
| Russia | 0.759 (0.014) | 0.915 (0.010) | 0.689 (0.017) | 0.773 (0.013) |
| Serbia | 0.828 (0.011) | 0.902 (0.009) | 0.728 (0.014) | 0.695 (0.015) |
| Slovakia | 0.852 (0.013) | 0.874 (0.013) | 0.737 (0.017) | 0.573 (0.024) |
| Sweden | 0.773 (0.021) | 0.823 (0.021) | 0.608 (0.026) | 0.691 (0.023) |
| Tajikistan | 0.789 (0.015) | 0.881 (0.012) | 0.630 (0.022) | 0.852 (0.013) |
| Turkey | 0.779 (0.017) | 0.854 (0.015) | 0.543 (0.026) | 0.778 (0.017) |
| Ukraine | 0.844 (0.011) | 0.873 (0.010) | 0.699 (0.015) | 0.632 (0.018) |
| Uzbekistan | 0.913 (0.006) | 0.934 (0.005) | 0.726 (0.014) | 0.883 (0.007) |
|  |  |  |  |  |

Note: All loadings are significant at p<0.05.

1. Table of trust means per country

|  | Pres. | Gov’t | Regional | Local | Parl. | Pol. Parties | Police | Armed Forces | Courts |
| --- | --- | --- | --- | --- | --- | --- | --- | --- | --- |
| Albania | 3.009 | 2.595 | 2.733 | 2.806 | 2.535 | 2.419 | 3.249 | 3.219 | 2.325 |
| Armenia | 2.680 | 2.565 | 2.673 | 2.827 | 2.425 | 2.422 | 2.791 | 3.781 | 2.419 |
| Azerbaijan | 4.553 | 3.513 | 3.077 | 3.151 | 3.163 | 2.722 | 3.359 | 4.216 | 3.048 |
| Belarus | 3.430 | 3.339 | 3.282 | 3.234 | 3.390 | 2.784 | 3.261 | 3.555 | 3.415 |
| Bosnia | 2.236 | 2.193 | 2.243 | 2.363 | 2.232 | 2.128 | 2.982 | 2.907 | 2.437 |
| Bulgaria | 2.665 | 2.478 | 2.488 | 2.653 | 2.138 | 1.966 | 2.825 | 2.904 | 2.158 |
| Croatia | 3.213 | 1.904 | 2.255 | 2.267 | 1.880 | 1.807 | 3.118 | 3.313 | 2.211 |
| Czech Rep | 3.348 | 2.426 | 2.782 | 3.071 | 2.314 | 2.172 | 3.092 | 3.232 | 2.718 |
| Estonia | 3.628 | 2.899 | 3.363 | 3.404 | 2.748 | 2.573 | 3.840 | 3.958 | 3.283 |
| France | 2.365 | 2.381 | 3.048 | 3.406 | 2.652 | 2.038 | 3.308 | 3.444 | 2.782 |
| Georgia | 3.414 | 3.236 | 3.246 | 3.213 | 3.087 | 2.615 | 3.657 | 3.701 | 3.029 |
| Germany | 3.329 | 2.724 | 3.013 | 3.205 | 2.858 | 2.396 | 3.802 | 3.438 | 3.634 |
| Gr. Britain | 3.478 | 2.338 | - | 2.640 | 2.436 | 2.295 | 3.654 | 4.161 | 3.255 |
| Hungary | 3.177 | 2.945 | - | 3.302 | 2.777 | 2.271 | 3.043 | 2.985 | 3.049 |
| Italy | 3.053 | 2.235 | 2.566 | 2.693 | 2.305 | 1.954 | 3.702 | 3.631 | 2.728 |
| Kazakhstan | 4.072 | 3.515 | 3.416 | 3.355 | 3.455 | 3.063 | 3.033 | 3.507 | 3.046 |
| Kosovo | 2.594 | 2.410 | - | 2.790 | 2.547 | 2.496 | 2.818 | 3.788 | 2.525 |
| Kyrgyzstan | 2.412 | 2.461 | 2.728 | 2.876 | 2.556 | 2.538 | 2.724 | 3.303 | 2.234 |
| Latvia | 2.735 | 2.133 | - | 2.842 | 1.987 | 1.862 | 3.169 | 3.260 | 2.751 |
| Lithuania | 3.714 | 2.073 | 2.458 | 2.559 | 2.005 | 2.052 | 3.001 | 3.129 | 2.363 |
| Macedonia | 2.581 | 2.412 | - | 2.565 | 2.302 | 2.118 | 2.976 | 3.137 | 2.248 |
| Moldova | 2.342 | 2.366 | 2.499 | 2.633 | 2.287 | 2.276 | 2.528 | 3.026 | 2.282 |
| Mongolia | 2.899 | 2.817 | 2.987 | 3.077 | 2.725 | 2.346 | 3.319 | 3.724 | 2.655 |
| Montenegro | 3.268 | 3.128 | - | 3.123 | 3.053 | 2.793 | 3.282 | 3.374 | 3.139 |
| Poland | 3.079 | 2.798 | 3.027 | 3.080 | 2.731 | 2.506 | 3.348 | 3.461 | 3.090 |
| Romania | 1.803 | 1.594 | 2.216 | 2.514 | 1.643 | 1.594 | 2.855 | 3.139 | 1.998 |
| Russia | 3.432 | 3.116 | 2.865 | 2.794 | 2.736 | 2.411 | 2.648 | 3.244 | 2.677 |
| Serbia | 2.641 | 2.125 | 2.272 | 2.294 | 2.089 | 1.870 | 2.948 | 3.136 | 2.331 |
| Slovakia | 3.074 | 2.501 | 2.689 | 2.873 | 2.406 | 2.323 | 3.048 | 3.306 | 2.659 |
| Slovenia | 2.863 | 2.247 | - | 2.853 | 2.288 | 2.269 | 3.076 | 3.220 | 2.620 |
| Sweden | 3.441 | 3.536 | 3.265 | 3.379 | 3.598 | 3.085 | 4.013 | 3.344 | 3.985 |
| Tajikistan | 4.639 | 4.304 | 4.133 | 4.017 | 3.968 | 3.434 | 3.612 | 3.987 | 3.835 |
| Turkey | 3.595 | 3.395 | 3.491 | 3.452 | 3.334 | 2.994 | 3.697 | 3.799 | 3.429 |
| Ukraine | 2.504 | 2.367 | 2.492 | 2.653 | 2.205 | 2.212 | 2.313 | 2.991 | 2.116 |
| Uzbekistan | - | 4.401 | 4.337 | 4.283 | 4.369 | 4.017 | 3.918 | 4.343 | 4.023 |

4) EFA output per country


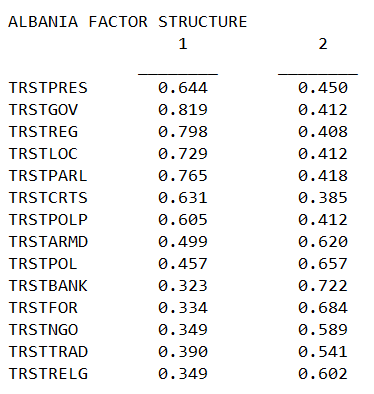

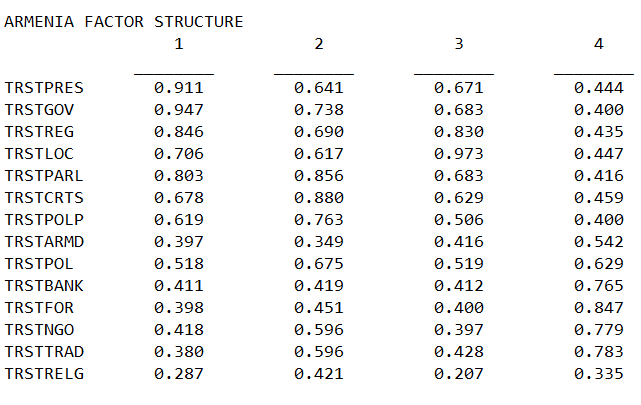


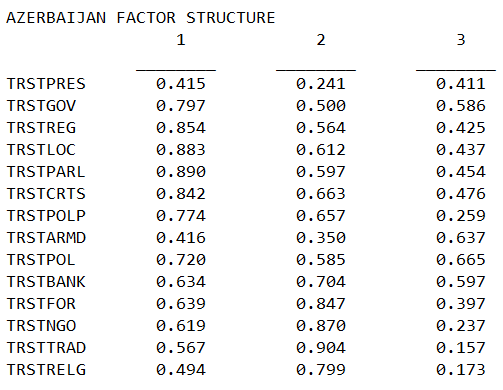


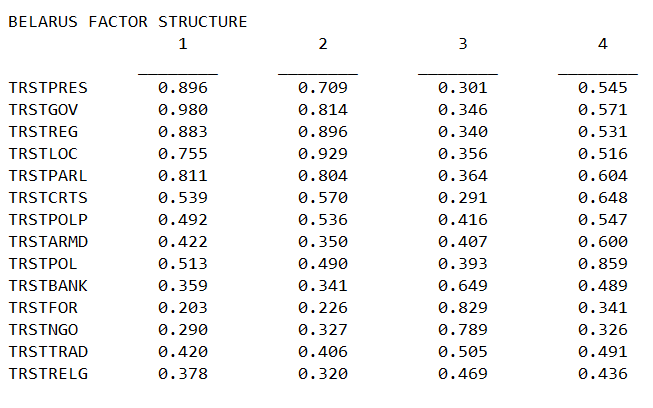


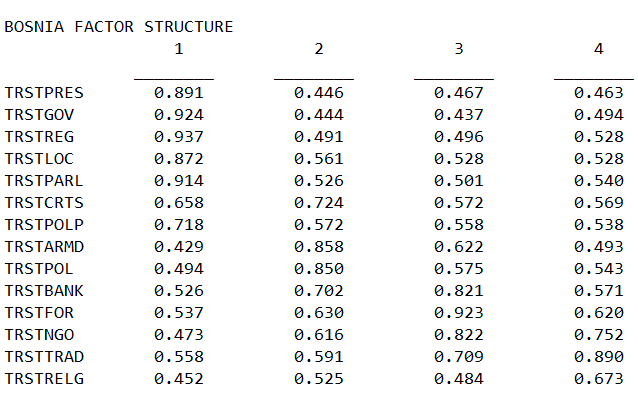


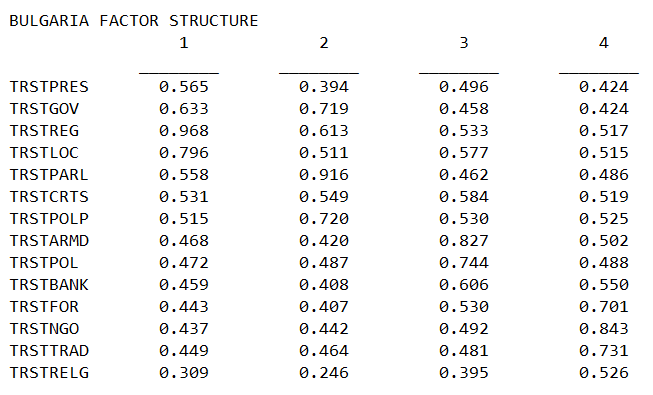


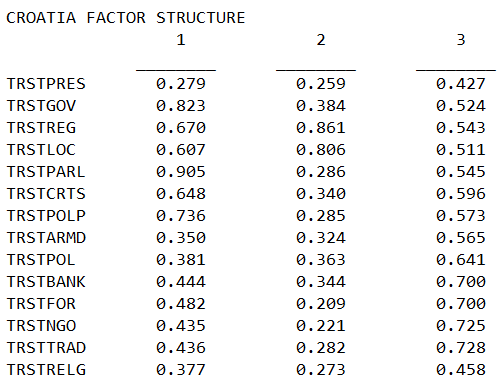


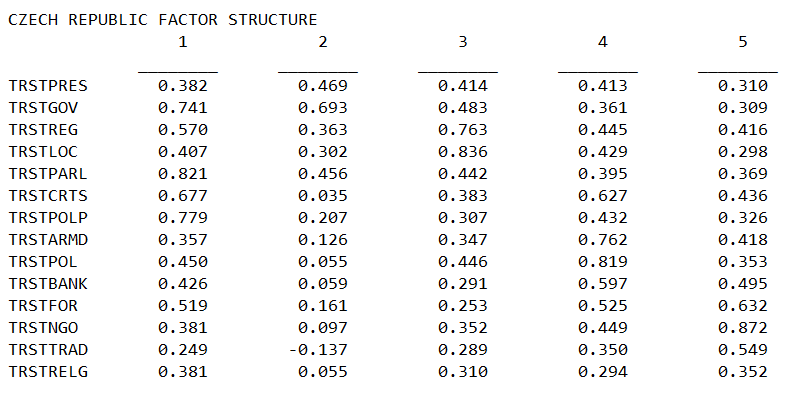


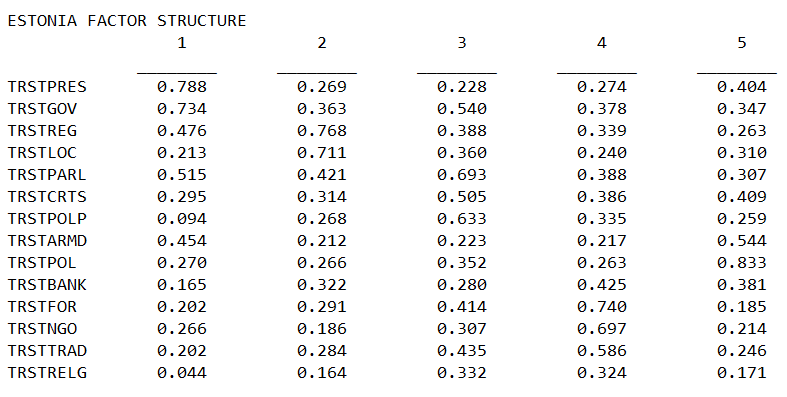


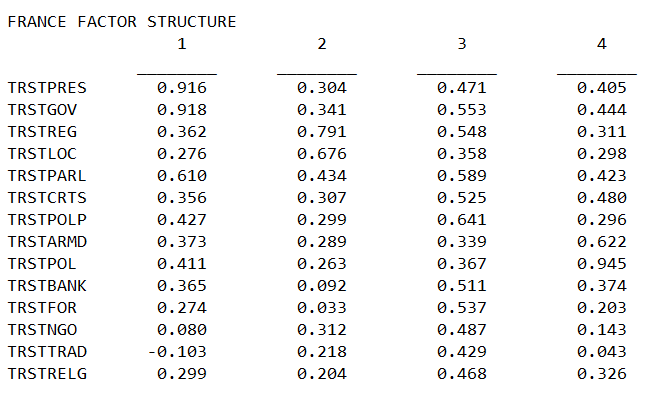


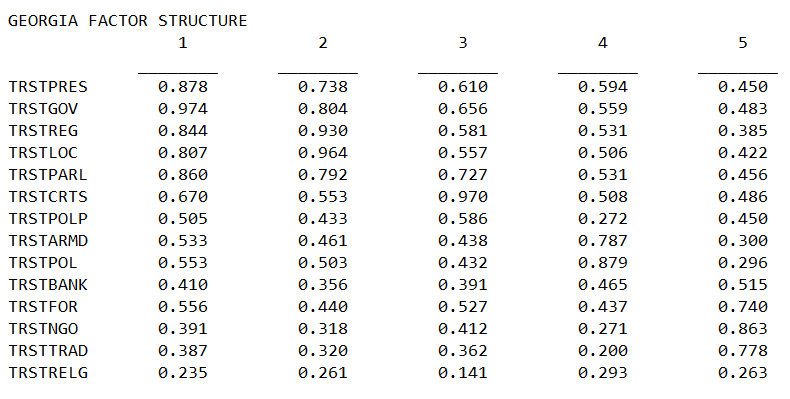


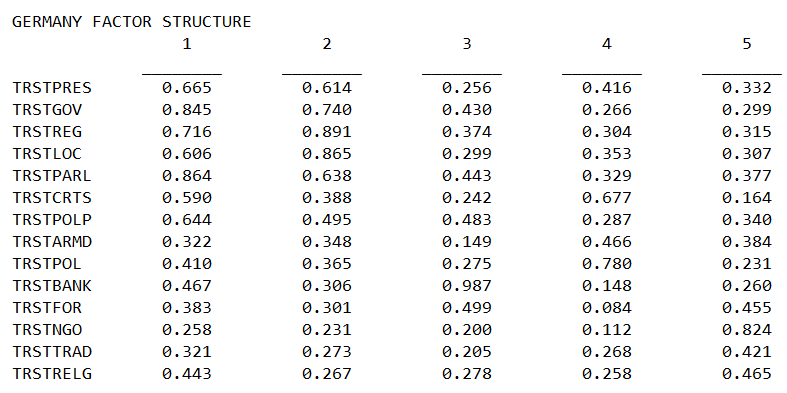


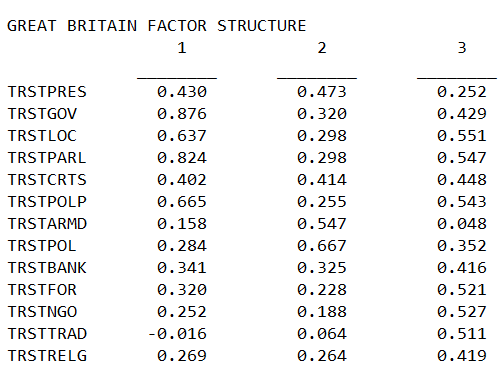


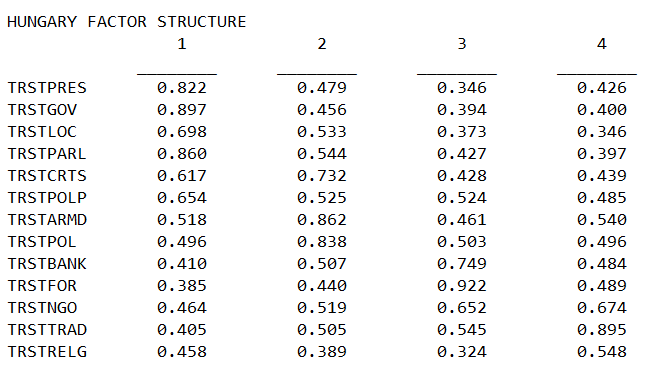


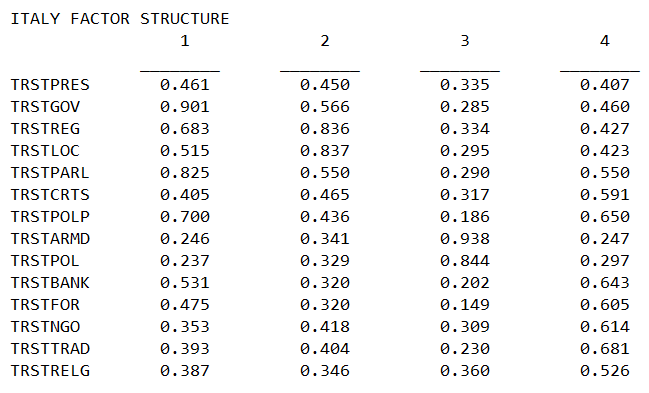


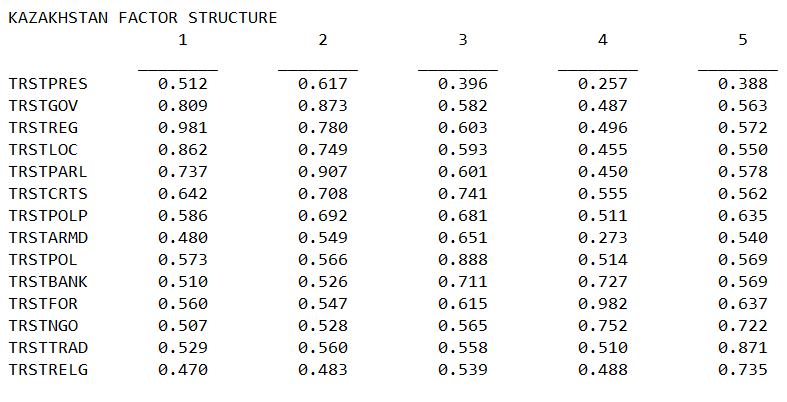


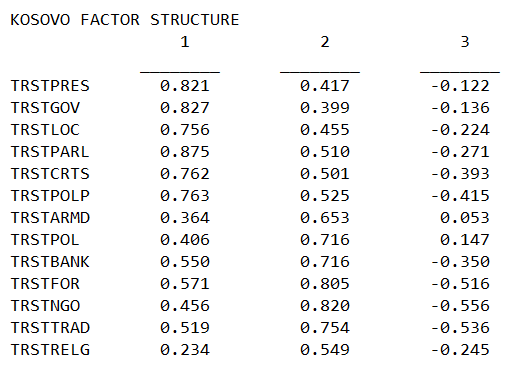


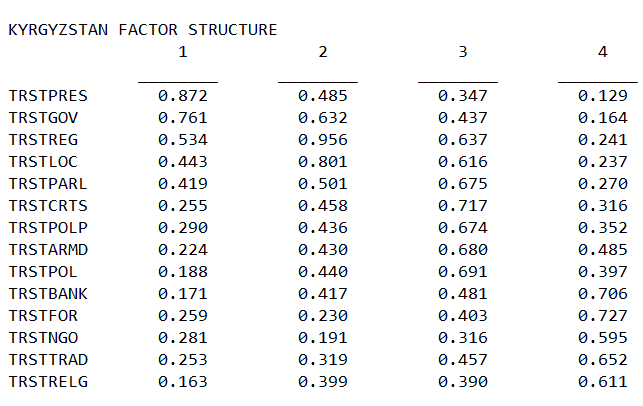


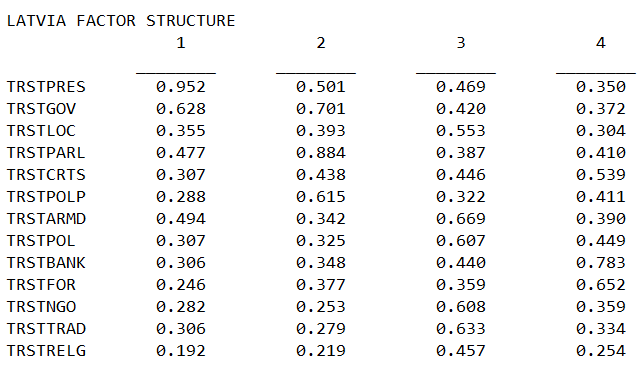


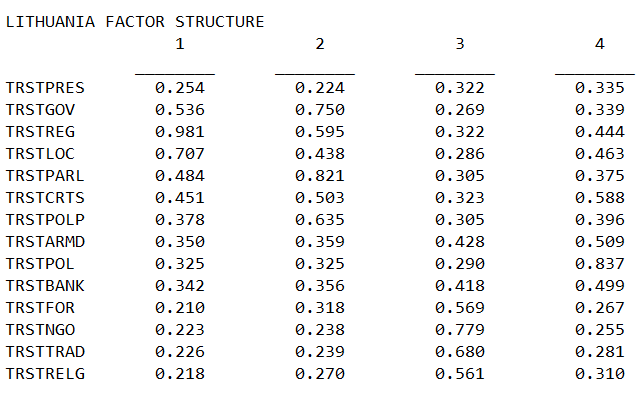


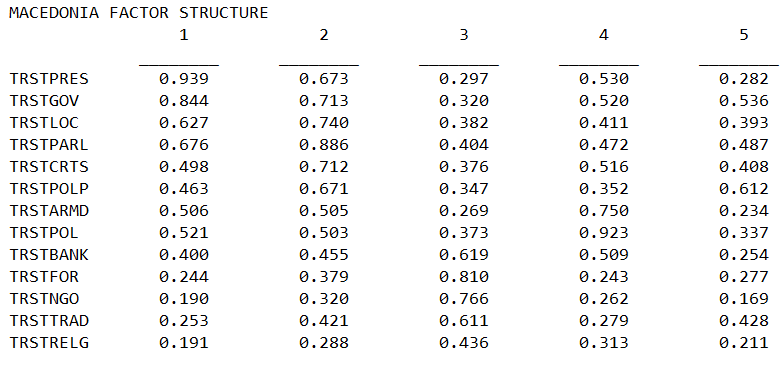


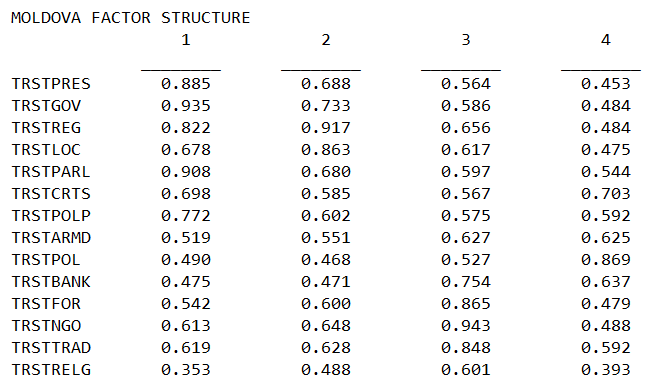


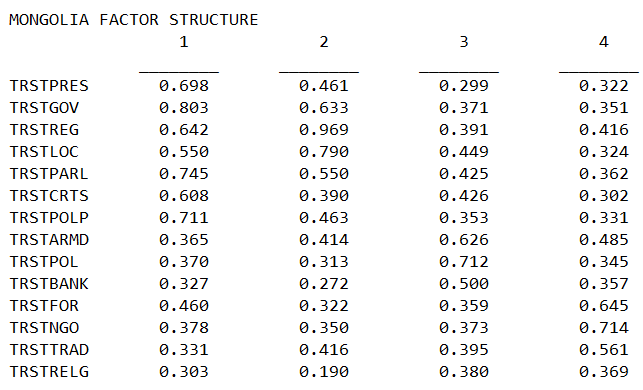


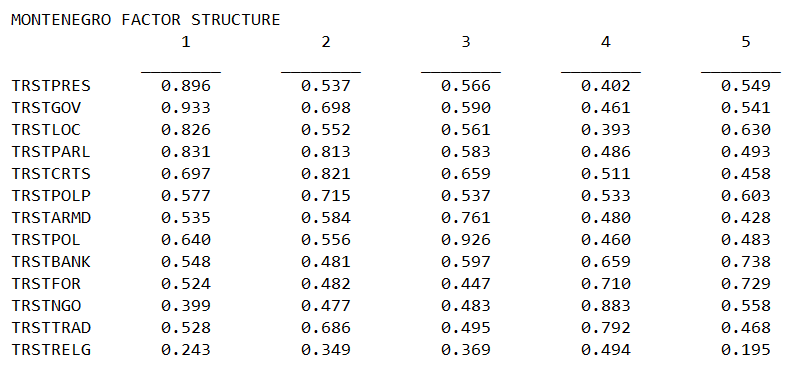


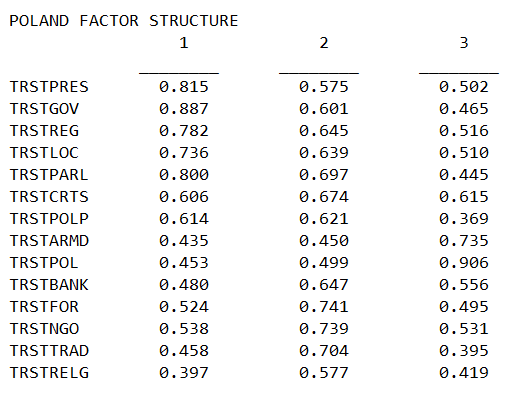


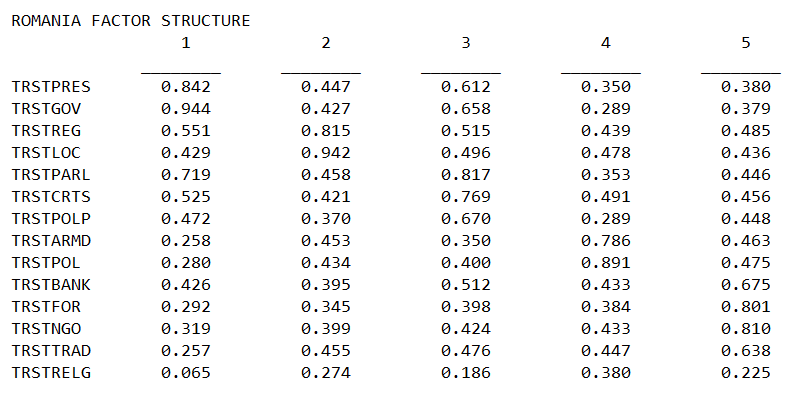


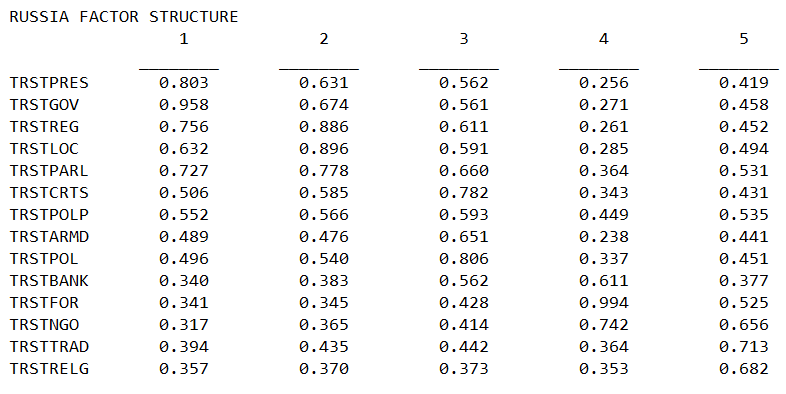


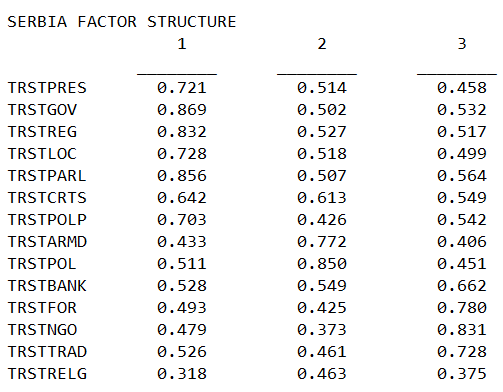


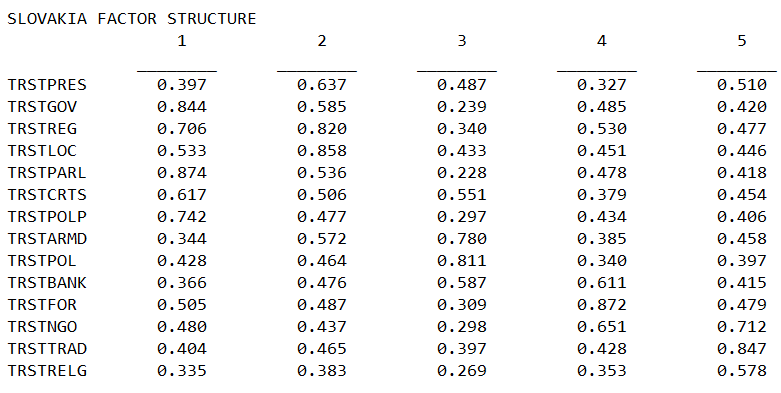


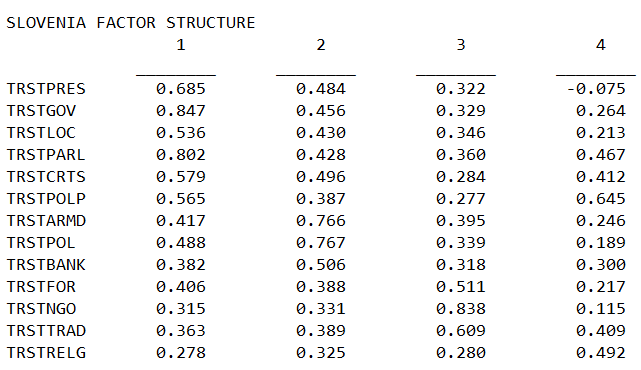


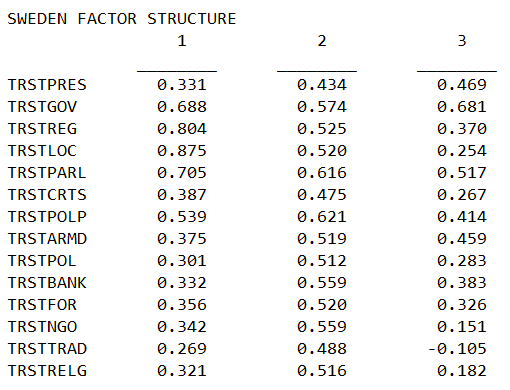


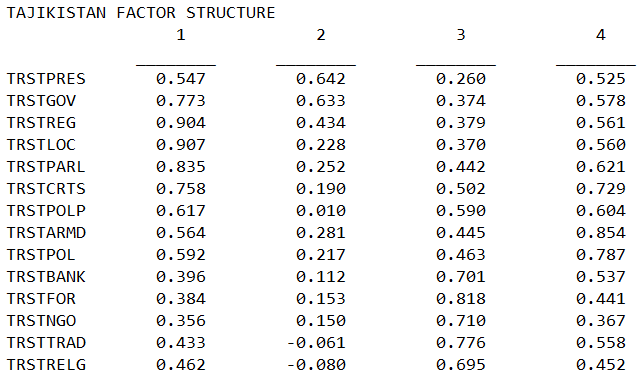


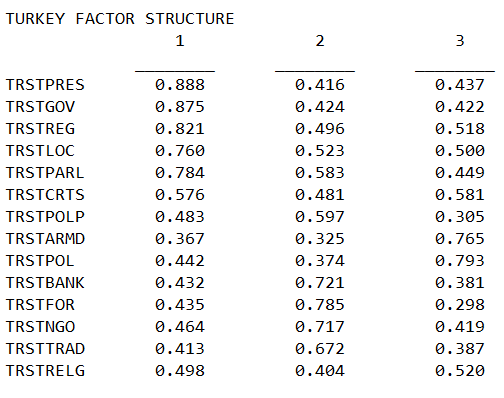


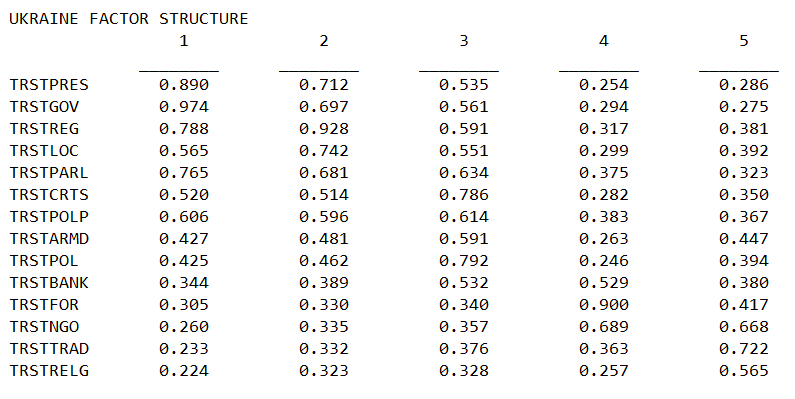


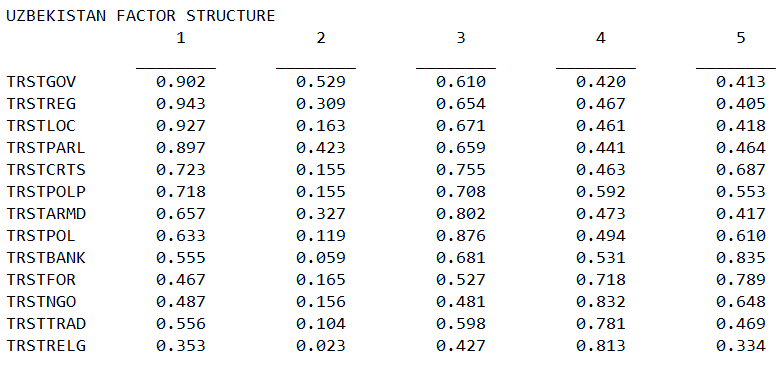


5) Mplus input code for CFA

Title: CFA for distant versus local model: Croatia

Data:

File is LITStrustnomiss.dta.dat ;

Variable:

Names are

country GENTRUST TRSTPRES TRSTGOV TRSTREG TRSTLOC TRSTPARL TRSTCRTS

TRSTPOLP TRSTARMD TRSTPOL TRSTBANK TRSTFOR TRSTNGO TRSTTRAD TRSTRELG;

Missing are all (-9999) ;

USEVARIABLES ARE TRSTGOV TRSTREG TRSTLOC TRSTPARL TRSTPOLP;

USEOBSERVATIONS ARE COUNTRY EQ 42;

Analysis:

Type IS MISSING;

Estimator = ML;

MODEL:

POLIT BY TRSTGOV TRSTPARL TRSTPOLP TRSTREG TRSTLOC;

TRSTREG WITH TRSTLOC;

OUTPUT:

SAMPSTAT STDYX MODINDICES TECH4;

Title: CFA for simple model: Great Britain

Data:

File is LITStrustnomiss.dta.dat ;

Variable:

Names are

country GENTRUST TRSTPRES TRSTGOV TRSTREG TRSTLOC TRSTPARL TRSTCRTS

TRSTPOLP TRSTARMD TRSTPOL TRSTBANK TRSTFOR TRSTNGO TRSTTRAD TRSTRELG;

Missing are all (-9999) ;

USEVARIABLES ARE TRSTGOV TRSTLOC TRSTPARL TRSTPOLP;

USEOBSERVATIONS ARE COUNTRY EQ 66;

Analysis:

Type IS MISSING;

Estimator = ML;

MODEL:

POLLOC BY TRSTGOV TRSTLOC TRSTPARL TRSTPOLP;

OUTPUT:

SAMPSTAT STDYX MODINDICES TECH4;

Title: CFA for political versus protective trust: Russia

Data:

File is LITStrustnomiss.dta.dat ;

Variable:

Names are

country GENTRUST TRSTPRES TRSTGOV TRSTREG TRSTLOC TRSTPARL TRSTCRTS

TRSTPOLP TRSTARMD TRSTPOL TRSTBANK TRSTFOR TRSTNGO TRSTTRAD TRSTRELG;

Missing are all (-9999) ;

USEVARIABLES ARE TRSTGOV TRSTPARL TRSTPOLP TRSTPOL TRSTARMD;

USEOBSERVATIONS ARE COUNTRY EQ 141;

Analysis:

Type IS MISSING;

Estimator = ML;

MODEL:

POLLOC BY TRSTGOV TRSTPARL TRSTPOLP;

ARMPOL BY TRSTPOL TRSTARMD;

OUTPUT:

SAMPSTAT STDYX MODINDICES TECH4;
